# Endogenous but not sensory-driven activity controls migration, morphogenesis and survival of adult-born juxtaglomerular neurons in the mouse olfactory bulb

**DOI:** 10.1007/s00018-023-04753-4

**Published:** 2023-03-18

**Authors:** Kaizhen Li, Katherine Figarella, Xin Su, Yury Kovalchuk, Jessika Gorzolka, Jonas J. Neher, Nima Mojtahedi, Nicolas Casadei, Ulrike B. S. Hedrich, Olga Garaschuk

**Affiliations:** 1 Department of Neurophysiology, Institute of Physiology, University of Tübingen, Tübingen, Germany; 2 German Center for Neurodegenerative Diseases (DZNE), Tübingen, Germany; 3 Department of Cellular Neurology, Hertie Institute for Clinical Brain Research, University of Tübingen, Tübingen, Germany; 4 Institute of Medical Genetics and Applied Genomics, University of Tübingen, Tübingen, Germany; 5 NGS Competence Center Tübingen, Tübingen, Germany; 6 Department of Neurology and Epileptology, Hertie Institute for Clinical Brain Research, University of Tübingen, Tübingen, Germany; 7 Present Address: Department of Physiology, University of Bern, Bern, Switzerland

**Keywords:** Adult neurogenesis, Potassium channels, Olfactory bulb, Neuronal development, Endogenous activity, Spontaneous calcium transients, pCREB, Migration, Differentiation, Survival

## Abstract

The development and survival of adult-born neurons are believed to be driven by sensory signaling. Here, in vivo analyses of motility, morphology and Ca^2+^ signaling, as well as transcriptome analyses of adult-born juxtaglomerular cells with reduced endogenous excitability (via cell-specific overexpression of either Kv1.2 or Kir2.1 K^+^ channels), revealed a pronounced impairment of migration, morphogenesis, survival, and functional integration of these cells into the mouse olfactory bulb, accompanied by a reduction in cytosolic Ca^2+^ fluctuations, phosphorylation of CREB and pCREB-mediated gene expression. Moreover, K^+^ channel overexpression strongly downregulated genes involved in neuronal migration, differentiation, and morphogenesis and upregulated apoptosis-related genes, thus locking adult-born cells in an immature and vulnerable state. Surprisingly, cells deprived of sensory-driven activity developed normally. Together, the data reveal signaling pathways connecting the endogenous intermittent neuronal activity/Ca^2+^ fluctuations as well as enhanced Kv1.2/Kir2.1 K^+^ channel function to migration, maturation, and survival of adult-born neurons.

**Supplementary Information:**

The online version contains supplementary material available at 10.1007/s00018-023-04753-4.

## Introduction

The rodent olfactory bulb (OB) is a highly plastic brain region receiving new neurons throughout life. Cumulative evidence revealed an important role of these cells for the fine-tuning of odor perception/discrimination, facilitation of task-dependent pattern separation, learning and memory [1–6]. Generated in the subventricular zone (SVZ) of the lateral ventricle [7, 8], adult-born cells migrate along the rostral migratory stream (RMS) into the OB and differentiate into local GABAergic interneurons: granule cells (GCs) and juxtaglomerular cells (JGCs) [9]. Many molecules including GABA, glutamate, dopamine, serotonin, BDNF, and cAMP response element-binding protein (CREB) influence adult OB neurogenesis [10–14]. Yet, the exact mechanisms underlying the migration, maturation, and incorporation of adult-born neurons into the existent OB circuitry remain unclear.

Many studies point to the key role of sensory experience [15–18]. Indeed, adult-born cells respond to odorants right after their appearance in the OB [19, 20], develop larger and more complex dendritic trees in the odor-enriched environment [21], and manipulations increasing sensory-driven activity like odor enrichment [22, 23], odor discrimination training [5, 24] or olfactory learning [25] increased the survival and integration of adult-born cells. Conversely, manipulations reducing sensory-driven activity like naris occlusion [16, 26–28], naris cauterization and benzodiazepine treatment [29], knocking-out olfactory receptors in olfactory sensory neurons [30] or their axotomy [31] were reported to decrease survival and integration of adult-born neurons.

Alternatively, the endogenous activity might be of great importance. An indirect support for this assumption comes from the fact that adult-born JGCs developing in sensory-deprived bulbs had normal dendritic morphology and dynamics [32] and the apoptosis of adult-born OB neurons was induced by the chemogenetic activation of higher-order odor-processing brain areas [33], synapsing on these cells. However, the strong and cell-specific reduction of the excitability of adult-born GCs via expression of a nonrectifying variant of the Kir2.1 K^+^ channel did not affect the initial stages of their development. The cells successfully migrated into the OB, survived there for 2 weeks and developed normal synaptic contacts and spines [34]. Yet, many of them died later, at 4 weeks of age. Using a similar approach in neonatally-born GCs confirmed that endogenous activity is not required for their tangential (in RMS) or radial (towards the OB surface) migration but is needed for normal positioning and survival of GCs [35]. Of note, the same experimental protocol did not impact the positioning or survival of JGCs, thus revealing a striking difference between the two cell populations.

Moreover, while adult-born GCs migrate straight to their final destinations and integrate therein, adult-born JGCs (abJGCs) first enter the 3–4 weeks long pre-integration phase [36]. This phase, during which abJGCs undergo a millimeter-long lateral migration [36], extensively grow and prune their dendritic trees [19, 37] and exhibit ongoing endogenous activity [38] is unique to abJGCs and largely unexplored. Therefore, in the current study we have tested the role of the endogenous activity for migration, morphogenesis, and survival of abJGCs during the pre-integration phase. To do so, we genetically suppressed their excitability and used longitudinal in vivo two-photon imaging to monitor their developmental history. To understand the molecular pathways involved, we analyzed the transcriptome of adult-born cells right after their arrival into the OB.

## Materials and methods

### Mouse models

Three- to four-month-old C57BL/6 mice of either sex were used in this study and were assigned randomly to control and test groups. Animals were kept in pathogen-free conditions at 22 °C, 60% air humidity, 12-h light–dark cycle with ad libitum access to food and water. Females stayed in groups of 3–5 mice, males were kept individually. Mice of similar age were assigned randomly to control and test groups. Littermates were evenly distributed among experimental groups and each experimental group contained mice from different litters.

### Implantation of a cranial window

A chronic cranial window was implanted over the mouse OB as described previously [19, 36]. Mice were anesthetized by an intraperitoneal (i.p.) injection of ketamine/xylazine (80/4 μg/g of body weight (BW)). Anesthetic depth was monitored by toe pinches throughout the surgery and additional ketamine/xylazine (40/2 μg/g of BW) was injected when necessary. Dexamethasone (2 μg/g of BW) was administered intramuscularly before the surgery. Local anesthetic lidocaine (2%) was applied subcutaneously over the OB for 5–10 min before removing the scalp. The ointment was used to prevent dehydration of the mouse’s eyes. A circular cranial opening (3 mm in diameter) was made by repeated drilling over the two OB hemispheres. Small pieces of bone were removed with a sharp blade and tweezers. Extreme care was taken, not to damage any blood vessels on the surface of the OB. The opening was rinsed with a standard extracellular solution (composition in mM: 125 NaCl, 4.5 KCl, 26 NaHCO_3_, 1.25 NaH_2_PO_4_, 2 CaCl_2_, 1 MgCl_2,_ and 20 glucose; pH 7.4, when bubbled continuously with 95% O_2_ and 5% CO_2_) and covered with a glass coverslip (Ø 3 mm, Warner Instruments, Hamden, CT, USA). The gap between the edge of the coverslip and the skull was filled with cyanoacrylate glue and then strengthened by dental cement. During the surgery and until full recovery from anesthesia the mouse was kept on a heated plate. Postoperative care included an analgesic dose of carprofen (5 μg/g of BW) for 3 days subcutaneously and the antibiotic Enrofloxacin (1:100 v/v) in drinking water for consecutive 10 days. Mice were allowed to recover for at least 3–4 weeks and were subsequently examined for window clarity. Mice were singly housed after cranial window implantation on a 12 h light/dark cycle with food and water available ad libitum.

### Construction of viral vectors and production of viruses

All lentiviral vectors were based on the FUGW backbone [39]. The eGFP in the original FUGW plasmid was replaced by a Ca^2+^ indicator Twitch-2B at BamHI and EcoRI restriction sites to generate FUW-Twitch-2B [40]. Kv1.2wt-T2A and Kir2.1wt-T2A fragments were magnified by PCR (Phusion High-Fidelity PCR Kit, NEB) from pcDNA3-Kv1.2wt (human cDNA template) and Mgi-Kir2.1wt (mouse cDNA template, from Carlos Lois laboratory, Caltech) plasmids and were inserted into FUW-Twitch-2B between XbaI and BamHI restriction enzyme sites. To construct Lenti-Twitch-2B-T2A-H2B-mCherry, H2B-mCherry was inserted after Twitch-2B and T2A to enable nuclear-located expression of mCherry. The sequence of T2A used in this study is 5’-tgggccaggattctcctcgacgtcaccgcatgttagcagacttcctctgccctctccactgcctaccgg-3’.

The second generation of the virus packaging system was used in this study. Briefly, HEK-293 T cells were transiently transfected with plasmids encoding target genes (Lenti-Twitch-2B-T2A-H2B-mCherry, Lenti-Kv1.2-T2A-Twitch-2B, or Lenti-Kir2.1-T2A-Twitch-2B), plus viral packaging helper plasmids pMD2.G (plasmid #12259, Addgene) and psPAX2 (plasmid #12260, Addgene) as described previously [36]. The Lipofectamine 3000 reagent (Invitrogen) was used for transfection. HEK-293 T and 1F8 cells were cultured in Dulbecco’s Modified Eagle’s Medium (DMEM) with 10% heat-inactivated fetal bovine serum and 2 mM L-glutamine at 37 °C and 5% CO_2_ in air atmosphere. Cell lines were not authenticated. Mycoplasma contamination was controlled by regular PCR tests. To block the K^+^ channel overexpression-induced reduction of the lentivirus-producing capacity of HEK-293 T cells [41], K^+^ channel blocker Ba^2+^ (0.3 mM) was added to the cell culture medium. 48–72 h after transfection, cell culture supernatant containing viral particles was collected and concentrated by centrifugation at 135,000*g* at 4 °C for 2 h. Concentrated supernatants were resuspended with PBS and titrated in HEK-293 T cells. Titers of about 8 × 10^9^ virus particles per ml concentrated supernatant were used for the following experiments. Retroviral vector Mgi-Kir2.1mut encoding 3 dominant negative site mutations (G144A, Y145A, G146A; Carlos Lois laboratory, Caltech), was packaged in 1F8 cells derived from 293GPG cell line. The vector was derived from a Moloney leukemia virus with an internal promoter from the Rous sarcoma virus. 48 h after transfection, the cell culture supernatant containing the retroviral particles was harvested and concentrated by centrifugation at 135,000*g* at 4 °C for 6 h. After concentration, the pellet was resuspended with as little volume of ice-chilled PBS as possible. Titers above 10^8^ particles per ml supernatant were used for in vivo injections.

### Virus injection into the RMS

Animals with implanted cranial windows were anesthetized with ketamine/xylazine (80/4 μg/g of BW) and fixed in a stereotaxic frame. The ointment was used to prevent dehydration of the mouse’s eyes and 2% lidocaine was applied subcutaneously on top of the injection sites. The skin was carefully removed to expose the skull and two small cranial openings (~ 0.5 mm in diameter) were drilled at the following coordinates: anterior–posterior + 3.0 mm, medial–lateral ± 0.83 mm. Through an opening in each hemisphere ~ 0.8–1 μl of the virus-containing solution was stereotactically injected into the RMS at a depth of -2.95 ± 0.05 mm from the pial surface and a speed of 100 nL/min via a glass micropipette. Thereafter, a metal bar, required for head fixation during the subsequent imaging sessions, was fixed to the caudal part of the skull with dental cement. The other exposed parts of the skull were also covered with dental cement. The mice were returned to the home cage and carprofen (5 μg/g of BW) was injected subcutaneously for 3 subsequent days.

### In vivo two-photon imaging

Mice with implanted cranial windows were anesthetized with either isoflurane or the MMF (medetomidine 0.5 μg/g BW, midazolam 5.0 μg/g BW, fentanyl 0.05 μg/g BW) anesthesia and placed on a heating plate. Breathing rate and body temperature were monitored continuously using the animal monitoring system (AD Instruments, Sydney, Australia). The head of the mouse was fixed with the metal bar to the *X*–*Y* table, ensuring consistent positioning through imaging sessions. In vivo two-photon imaging was performed using an Olympus FV1000 system (Olympus, Tokyo, Japan) with a MaiTai Deep See Laser (Spectra-Physics, Mountain View, CA, USA) and a Zeiss 20 × water-immersion objective lens (NA 1.0, Carl Zeiss, Jena, Germany). Unless otherwise indicated, cells were imaged using the 890 nm excitation wavelength.

#### Imaging migration of adult-born JGCs

Mice were anesthetized with isoflurane (2% for induction, 0.8–1.0% for maintenance) and transferred into the imaging setup. The body temperature was kept at ~ 37 °C. Breathing rate was monitored during the whole imaging session and maintained at 110–140 breaths per minute by slightly adjusting the isoflurane concentration in O_2_. To measure the migration speed, the positions of adult-born neurons were monitored every 15 min for 4 h at 8 and 14 dpi according to the previously established protocol [36]. To create landmarks for single-cell tracking, we labeled blood vessels via i.p. injection of sulforhodamine B (0.1 ml/20 g BW, 1 mM in PBS, Sigma-Aldrich, St. Louis, USA). In addition, the same FOVs were re-imaged with the same imaging settings at 11, 25, and 45 dpi.

#### Recording spontaneous and odor-evoked Ca^2+^ transients of adult-born JGCs

The spontaneous Ca^2+^ transients were recorded in awake mice. Prior to imaging sessions, the mice were trained for head fixation for 10–12 days, as described in ref. [13]. Spontaneous Ca^2+^ transients of abJGCs were recorded at 12 dpi continuously for 2 min with a frame rate of 7–10 Hz. Twitch-2B was excited at 890 nm and the emitted light was split into 2 channels by a 515 nm dichroic mirror. The emission light of mCerulean3 was filtered with a 475/64 nm band-pass filter and the emission light of cpVenus^CD^ was filtered with a 500 nm long-pass filter. Note that all major characteristics of spontaneous activity (e.g., the fraction of spontaneously active cells, the maximum Twitch-2B ratio, the area under the curve, the fraction of time spent in the active state, etc.) stay constant between DPI 12 and 22 (fig. 5 in ref. [38]).

The odor-evoked responsiveness of abJGCs was measured at 20 dpi. Mice were anesthetized using the MMF anesthesia, the temperature was kept at ~ 37 °C and the breathing rate was ~ 140 breaths per minute during the whole imaging session. Note that under this anesthesia, neither fractions of odor-responding and non-responding, nor the fractions of reliably responding, non-reliably responding and nonresponding abJGCs differ between awake and anesthetized mice (Chi-Square test, p = 0.36 and 0.12, respectively; [13]). Odors were applied through a custom-built flow-dilution olfactometer, positioned in front of the mouse’s snout as described previously [42]. An odor mixture containing 2-hexanone, isoamyl acetate, and ethyl tiglate (purchased from Sigma-Aldrich, 0.6% of saturated vapor each) was applied as a 4-s-long pulse with an inter-pulse interval of at least 2 min. The odor delivery was not timed relative to respiration. Each cell was stimulated at least twice, as described in ref. [42].

### Odor deprivation (OD) and two-photon imaging of odor-deprived mice

Cranial window and virus injection were done using the protocol described above. Lenti-Twitch-2B-T2A-mCherry was injected in both hemibulbs. Unilateral naris closure was performed at 5 dpi. Nose plugs were constructed of a 2 mm polyethylene tube (0.58 mm inner diameter, 0.96 mm outer diameter, Portex, UK) and suture thread (size 3-0, Ethicon, Germany) as described previously [36]. Mice were anesthetized using the MMF anesthesia and the plug was accurately inserted into the nostril. Thereafter, mice received an antidote containing flumazenil (0.5 mg/kg BW, Fresenius, Germany) and atipamezole (2.5 mg/kg BW, Alfavet, Germany) and returned to their home cages. After naris occlusion, physiological conditions of experimental animals (e.g., breathing, body weight, and stability of nose plug) were monitored carefully every day. Two-photon imaging of odor-deprived mice was conducted under the MMF anesthesia, as described above.

### Immunohistochemistry

Mice were transcardially perfused with 4% paraformaldehyde (PFA) in PBS. The brains were removed and fixed in 4% PFA for 24 h at 4 °C, and then cryoprotected in 25% sucrose in PBS overnight at 4 °C. Next, the brains were embedded in Tissue Tek (Sakura, Zoeterwoude, Netherlands) and frozen at − 80 °C. The immunostaining was performed on free-floating sagittal cryoslices (thickness 30–50 μm) at room temperature. The sections were incubated in a blocking buffer containing 5% normal donkey serum (Jackson Immuno Research, Dianova) and 0.1% Triton-X 100 (Sigma, USA) in PBS for 1 h to prevent nonspecific background staining. After blocking, the sections were incubated with the primary antibodies diluted in the blocking buffer. The following primary antibodies were used: goat polyclonal antibody against GFP (Rockland 600-101-215, 1:1000), mouse monoclonal antibody against Kv1.2 (NeuroMab 75-008, 1:200), rabbit monoclonal antibody against pCREB (Cell Signaling 9198S, 1:400), mouse monoclonal antibody against NeuN (Millipore MAB377, 1:1000), rabbit polyclonal antibody against DCX (Abcam ab18723, 1:1000). After overnight incubation with primary antibodies at 4 °C, the sections were rinsed in PBS three times for 10 min each and incubated with secondary antibodies (2% BSA and 1% Triton-X 100 in PBS) for 2 h in the dark at room temperature. The secondary antibodies were as follows: donkey-anti-mouse or anti-rabbit IgG-conjugated Alexa Fluor 488 (A21202 or A21206, 1:1000), donkey-anti-goat IgG-conjugated Alexa Fluor 594 (A11058, 1:1000), donkey-anti-mouse IgG conjugated Alexa Fluor 680 (A10038, 1:1000), all purchased from Invitrogen (Grand Island, NY). Afterward, the sections were washed three times in PBS for 10 min, transferred to Superfrost Plus charged glass slides (Langenbrink, Emmendingen, Germany), and mounted in Vectashield (Vector Laboratories, USA) or ProLong Gold (Invitrogen) mounting medium. Immunostained slices were imaged using an Olympus Fluoview 300 laser scanning microscope (Olympus, Tokyo, Japan) coupled with a MaiTai mode-locked laser (Spectra Physics, Mountain View, CA, USA). Alexa Fluor 488 and 594 were excited at 800 nm and the emitted light was split by a 570 nm dichroic mirror and filtered with a 536/40 nm band-pass filter as well as a 570 nm long-pass filter. Alexa Fluor 680 was also excited at 800 nm and the signal was collected in the long-pass channel of a 670 nm dichroic mirror.

### RNA sequencing

Male 3-month-old mice were bilaterally injected into the RMS with either Lenti-Twitch-2B-T2A-H2B-mCherry, Lenti-Kv1.2-T2A-Twitch-2B, or Lenti-Kir2.1-T2A-Twitch-2B viruses and sacrificed at 9 dpi. Mice were deeply anesthetized by ketamine/xylazine (100/10 μg/g of BW) and transcardially perfused with 20 ml ice-cold PBS to empty blood vessels from the blood. Then mice were decapitated, and the olfactory bulbs were quickly dissected and transferred into an ice-cold 35 mm dish containing 0.5 ml dissection medium (Hank’s balanced salt solution containing 15 mM HEPES, 25 mM glucose, 0.4 mg/ml DNase I, and 80 U/ml RNAse inhibitor). Olfactory bulbs from two animals were pooled to assure the isolation of at least 3000 adult-born cells. Minced tissue was very gently homogenized in an ice-cold dissection medium and passed through a 70 µm cell strainer. The filtrate was spun for 10 min at 250 g in a refrigerated centrifuge at 4 °C. Pellet was resuspended in 37% Percoll and centrifuged for 30 min at 800 g and 4 °C. First, the upper myelin layer and then supernatant were removed, the pellet was resuspended in sorting buffer (dissection medium without DNase I) and centrifuged once again for 10 min at 800 g and 4 °C. Finally, isolated cells were resuspended in a sorting buffer and kept on ice until sorting. Adult-born cells expressing Twitch-2B were separated by fluorescence-activated cell sorting on a Sony SH800Z sorter (Sony Biotechnology Inc, Surrey, UK) immediately after staining samples with propidium iodide (PI). Single cells were selected based on FSC-W/FSC-H gating, dead cells were excluded based on the PI-signals and transduced cells were identified by their Twitch-2B signal (using a 450/50 bandpass filter). Single Twitch-2B-expressing cells were sorted directly into Eppendorf tubes containing 10 µl sorting buffer, immediately frozen in liquid nitrogen, and kept at − 80 °C until the libraries for RNA-sequencing analysis were prepared.

The synthesis of the cDNA was performed using the SMART-Seq v4 Ultra Low Input RNA Kit (Takara Bio). 3000 to 4500 frozen sorted cells were lysed using a concentration of 100 cells per µl of lysis buffer. Lysis was performed by resuspending the cells by pipetting and incubating for 5 min at room temperature. First-strand cDNA synthesis was performed using 5 µl of cDNA for 90 min at 42 °C. Amplification of the full-length double-strand cDNA was monitored by qPCR and was stopped at 17 PCR cycles during the linear amplification phase. The resulting cDNA presented a fragment size distribution of 1500 up to 5000 bp on the Bioanalyzer High Sensitivity DNA Kit (Agilent) and a concentration above 300 pg/µl measured with Qubit dsDNA HS fluorometric quantification (ThermoFisher Scientific). Next-generation sequencing (NGS) libraries were prepared using 150 pg of cDNA input in the Nextera XT DNA Library Preparation Kit (Illumina), followed by 12 cycles of PCR. Final libraries had a mean fragment size of 370 bp on the Bioanalyzer, a concentration > 5 ng/µl, and a molarity of > 30 nmol/l measured with Qubit. Libraries were sequenced as single reads (75 bp read length) on a NextSeq500 (Illumina) with a depth of > 20 million reads. Library preparation and sequencing procedures were performed by the same individual and we chose a design, aimed to minimize technical batch effects.

### Quantitative real-time PCR

Reverse transcribed RNA from sorted cells was generated by SMART-Seq v4 Ultra Low Input RNA Kit (Takara Bio). Real-time PCR was performed using a Fast SYBR green PCR Master Mix (Applied Biosystems) and the QuantStudio™ 5 equipment (Applied Biosystems, Germany). Specific primers (Supplementary Table S3) were designed using the NCBI tool primer BLAST selecting those sequences that span an exon-exon junction to avoid amplification of any contaminating genomic DNA. Three biological samples were determined in triplicate. Single-product amplification was confirmed by running the melting curves. Input quantities were normalized to those for glyceraldehyde 3-phosphate dehydrogenase (*Gapdh*) and the relative mRNA expression was estimated by the efficiency corrected method [43].

### Electrical recordings

20 days after virus injection into the RMS (20 dpi), 250 µm thick sagittal OB slices were cut on Microm HM 650 V vibratome (Thermo Fisher Scientific, Dreieich, Germany). After an incubation period of up to 1 h, slices were transferred into the experimental setup and perfused with the extracellular solution containing (in mM): 125 NaCl, 3.5 KCl, 26 NaHCO_3_, 1.25 NaH_2_PO_4_, 2 CaCl_2_, 1 MgCl_2_, and 20 glucose, pH 7.4 when bubbled with 95% O_2_ and 5% CO_2_. Experiments were carried out at ~ 33 °C. Twitch-2B-positive control or Kv1.2/Kir2.1 overexpressing abJGCs were recorded in whole-cell configuration using an EPC-10 patch-clamp amplifier (HEKA, Lambrecht, Germany). The intracellular pipette solution contained (in mM): K-Gluconate 140, NaCl 10, HEPES 10, EGTA 0.2, Mg-ATP 4, Na-GTP 0.4, Alexa Fluor 594 0.05, pH = 7.3. Current and voltage traces were acquired at a 20 kHz sampling rate. For current-clamp recordings, currents of different polarity and amplitude (usually from − 40 pA to + 100 pA, 100 or 200 ms long) were injected stepwise to assess the cell’s firing ability. The holding current was adjusted to maintain basal membrane potential at − 70 mV.

### Analyses

#### Calculation of the migration speed of abJGCs

The migration speed of abJGCs was analyzed as described previously [36]. The 3D image stacks acquired during the consecutive imaging sessions (containing both abJGCs and blood vessels) were aligned offline using the blood vessel pattern as an anatomical landmark. Each cell received an identification number, and its position (the center of the cells’ soma) was identified in each of 17 stacks (0–4 h, 15 min inter-stack interval), enabling the reconstruction of the cell trajectory. Next, the X–Y coordinates of each cell’s position were read out using the Fluoview 3.0 Viewer (Olympus). For Z-axis coordinates, the depth of the cells was the relative depth from the dura. With 3D coordinates for each position point, migration distance (D) between the two points in space was calculated according to the following formula:

$$D = \sqrt {\left( {X1 - X0} \right)^{2} + \left( {Y1 - Y0} \right)^{2} + \left( {Z1 - Z0} \right)^{2} }$$

where (X1, Y1, Z1) and (X0, Y0, Z0) were coordinates of the cells’ position in the current and the immediately preceding stacks, respectively. Migration speed was defined as the translocation of the cells’ soma between the two consecutive time points divided by the respective time interval (µm per 15 min). Because the step size of the acquired stacks was 2 μm, a cell was considered moving if the translocation of the cells’ soma between the two consecutive time points was more than 4 μm.

#### Analyses of spontaneous Ca^2+^ transients

Data analysis was performed offline with ImageJ and a custom-made routine in Matlab (R2016b, The MathWorks, United States). Circular regions of interest (ROIs) were manually drawn within the soma of each cell. A fluorescence trace for each cell was obtained by averaging all pixels within the ROI. The background signal was obtained from the ROI of a comparable size devoid of fluorescent processes and located near the cell of interest. The image stack was separated into 2 substacks for mCerulean3 and cpVenus^CD^ channels, respectively. The fluorescence traces were calculated separately for both substacks and filtered using a lowpass Butterworth infinite impulse response filter with a cut-off frequency of 0.6 Hz. The Twitch-2B ratio signal was calculated using the formula:

$$Ratio = \frac{{F \, \left( {cpVenus^{CD} } \right)Soma - F \, \left( {cpVenus^{CD} } \right)\,Background}}{{F \, \left( {m\,Cerulean3} \right)Soma - F \, \left( {m\,Cerulean3} \right)\,Background}}$$

Thereafter, the traces were imported into Matlab to analyze the following parameters: basal and maximum Twitch-2B ratio and the area under the curve normalized to the total recording time (AUC/sec, [38]). The basal ratio was the mean of the lowest 10% data points in the histogram of each individual trace. The value for the maximum ratio was calculated as follows: the filtered traces were processed by a sliding average algorithm with a window size of 1.5 s to determine the maximum ratio (maximum average value). Parameters of Ca^2+^ fluctuations were also analyzed in Matlab. To compare experimental and control groups, the fluctuations in Ca^2+^ signals were detected using the mid-reference level crossing approach (function midcross in Matlab) and the numbers of crossing points, passing through the mid-reference level, per trace were counted. The Gaussian mixture model [44] was used to explore and detect the naturally existing clusters among the counted numbers of crossing points. The correct number of clusters has been estimated using the Bayesian information criterion [45]. Assigned cluster labels (i.e., with and without fluctuations in Ca^2+^ signals) have been used to calculate the fraction of each cluster in a given experimental group. Subsequently, these fractions were compared statistically using ANOVA.

#### Analyses of odor-evoked responsiveness of adult-born JGCs

The odor-evoked Ca^2+^ transients of individual neurons were detected with a custom-written Igor Pro routine (WaveMetrics Inc., OR 97035 USA). First, background fluorescence, measured in the neuropil surrounding the abJGCs, was subtracted as described above and the Twitch-2B ratio signals were calculated and expressed as relative Twitch-2B ratio changes (ΔR/R). For automatic detection of responding cells, all ΔR/R traces were smoothed with a binomial filter (time window 0.3 s). Each smoothed trace was subtracted from the original ΔR/R trace, resulting in the “baseline noise” trace. ΔR/R transients were automatically detected with a template-matching algorithm, taking into account their sharp rise. A ΔR/R change was recognized as an odor-evoked Ca^2+^ transient if its amplitude was three times larger than the standard deviation of the corresponding baseline noise.

#### Sholl analyses

The dendritic morphology of abJGCs was analyzed both in vivo and in situ. The three-dimensional stacks were imported into Neuromantic software (https://www.reading.ac.uk/neuromantic/body_index.php) for 3D reconstruction. Neuronal morphology was manually traced to obtain accurate reconstructions. Digitally reconstructed neurons were imported into Image J and analyzed with the Simple Neurite Tracer plugin. The following morphological parameters were read out: the number of dendritic branches and the total dendritic branch length (TDBL). Sholl analysis was performed by counting the number of intersections between dendrites and centered on the soma concentric spheres with 10 μm radius increments [46].

#### Analyses of the survival rate

All FOVs including cells and blood vessels were imaged at 14, 25, and 45 dpi under the same settings. The size of each image stack was 635 µm × 635 µm × 200 µm (XYZ). To minimize the effects of cell migration, a safe margin amounting to 100 µm × 100 µm (XY) from the image border was introduced, and cells residing within the margin area were excluded from the analysis. By using blood vessels as a landmark, a cell was considered surviving if its soma was found at the same position in both image stacks (14 and 25 dpi, or 25 and 45 dpi). Based on the resolution of the microscope, an offset of ≤ 4 µm was tolerated.

#### Analyses of the pCREB expression level and quantification of the Kv1.2 expression

All brain slices were stained and imaged under the same conditions (i.e., antibody concentration, incubation time, laser power, photomultiplier voltage, etc.). Background-subtracted images were generated by estimating background noise from 5 negative control slices, which were stained with secondary antibodies only, and subtracting the median noise value from the original images. Each slice was simultaneously stained with anti-pCREB, anti-GFP (recognizes Twitch-2B), and anti-NeuN antibodies, with secondary antibodies conjugated with Alexa Fluor-488, 594, and 680, respectively. All images were processed using the following protocol in ImageJ: (i) generate 3 substacks for pCREB, GFP, and NeuN staining by splitting the original 3D stack; (ii) draw the ROIs for the abJGCs on the central slice (Z-axis) of the GFP (Twitch-2B) stack; (iii) find the corresponding frame (same depth) in the NeuN stack; (iv) subtract the median background noise value from the NeuN stack; (v) adjust the threshold of the image to highlight all NeuN positive regions and draw ROIs for NeuN-positive cells in the whole field of view; (vi) measure the fluorescence intensity of NeuN-positive mature neurons and GFP-positive abJGCs in the stack of pCREB; (vii) calculate the relative pCREB expression level as the intensity of pCREB fluorescence of each GFP-positive adult-born JGC divided by the median intensity of all NeuN-positive mature neurons located in the same field of view.

$${\text{Relative pCREB level }} = \frac{{{\text{FpCREB}}\left( {{\text{abJGCs}}} \right){-}{\text{ F}}\,background}}{{{\text{Median}}\left( {{\text{FpCREB}}\left( {{\text{NeuN}}^{ + } {\text{cells }}} \right){-}{\text{ F}}\,background} \right)}}$$

For quantification of the Kv1.2 expression, the background-subtracted images were generated as described above and a custom-written Matlab code was used to calculate separately the intensity of immunofluorescence in the cell somata and the surrounding neuropil. The relative Kv1.2 expression level was calculated as the ratio of the somatic fluorescence divided by the fluorescence of the surrounding neuropil.

#### Transcriptomic analyses

The read quality of RNA-seq data in fastq files was assessed with ReadQC (ngs-bits version 2019_09). Raw reads were filtered to remove sequencing adapters and for quality trimming using SeqPurge (ngs-bits version 2019_09). Filtered reads were aligned against the reference mouse genome of the Ensembl Mus Musculus GRCm38 using STAR (v2.7.0f), allowing gapped alignments to account for splicing. Low mapping quality reads or those mispairing or multi-mapping were removed using Samtools (v1.9) and visually inspected in the Integrative Genome Viewer (v2.4.19). A matrix of raw counts was built using subread (v1.6.4). Low-expressed transcripts were filtered out to minimize the false-positive rate. For each dataset, all transcripts with less than 1 count per million in at least two samples were excluded, leaving 11,611 genes for further differential expression analysis. Differentially expressed genes (DEGs) were identified using edgeR (v.3.24.3) with R (v3.5.2) (https://www.R-project.org/) following the standard workflow. With this method, the size of the library is corrected and differential expression is tested by using negative binomial generalized linear models [47]. Genes with an absolute > 2-times-fold change between the control and Kv1.2/Kir2.1 groups (885 genes) were considered as DEGs and imported into the online portal Metascape (https://metascape.org/gp/index.html#/main/step1) to run GO biological processes, cell components, molecular functions, and KEGG pathway enrichment analyses [48]. In this study, the focus was on signaling pathways downstream of pCREB as well as on the routes leading to CREB phosphorylation.

#### Electrical recordings

To calculate the threshold of an AP, the first derivative was analyzed and the membrane voltage that corresponds to a threefold S.D. of a baseline value was taken as a threshold [49]. The same results were obtained using two other methods for threshold definition: (a) rate-of-rise point of 50 kV/s [49] or (b) the first peak in the 3rd derivative [50] (not shown). The input resistance was measured in whole cell voltage-clamp configuration with cell membrane voltage set to − 70 mV by applying 500 ms-long hyperpolarizing pulses with amplitudes of 40–20 mV and measuring the respective steady-state currents. To determine resting membrane potentials, the I–V plots were linearly interpolated to measure the voltages at zero holding current. The data points for the I–V plots were obtained by clamping the cells at three different potentials − 110 mV, − 90 mV and − 70 mV and measuring the respective holding currents.

#### Statistical analyses

Statistical analyses were performed using the GraphPad Prism 9 software (GraphPad Software Inc, San Diego, California USA). The Shapiro–Wilk test was used to check the normality of data distribution within the data sets. *P* < 0.05 was considered statistically significant. In the case of normally distributed data, the parametric (i.e., Student’s t-test or ANOVA followed by Tukey's multiple comparison test) otherwise nonparametric (i.e., Mann–Whitney U test or Kruskal–Wallis test followed by Dunn's multiple comparisons test) tests were used. For Sholl analysis, we used the generalized mixed-effects model with the Poisson family (R programming language [51] with lme4 package [52]). P-values were obtained using the lmerTest package, which estimates p-values based on Satterthwaite approximation [53]. Post-hoc comparisons were done using the lsmeans package [54]. Box plots were used to present 5 parameters of the dataset: 10th, 25th percentile, median, 75th, and 90th percentile. Unless otherwise indicated, the error bars represent median ± IQR (interquartile range).

## Results

### Alteration of endogenous but not sensory-driven activity impaired the lateral migration of adult-born cells

To suppress the excitability of adult-born cells, we virally overexpressed in abJGCs either voltage-gated Kv1.2 channels or inwardly rectifying Kir2.1 channels [55]. Overexpression of Kir2.1 channels hyperpolarizes the resting cells but once the spike threshold is reached, the channels close and contribute negligibly to the spike waveform [56, 57]. Voltage-gated Kv1.2 channels are closed at rest, but their overexpression is expected to strengthen the cell’s hyperpolarization after the action potential (AP) firing.

**Fig. 1:**
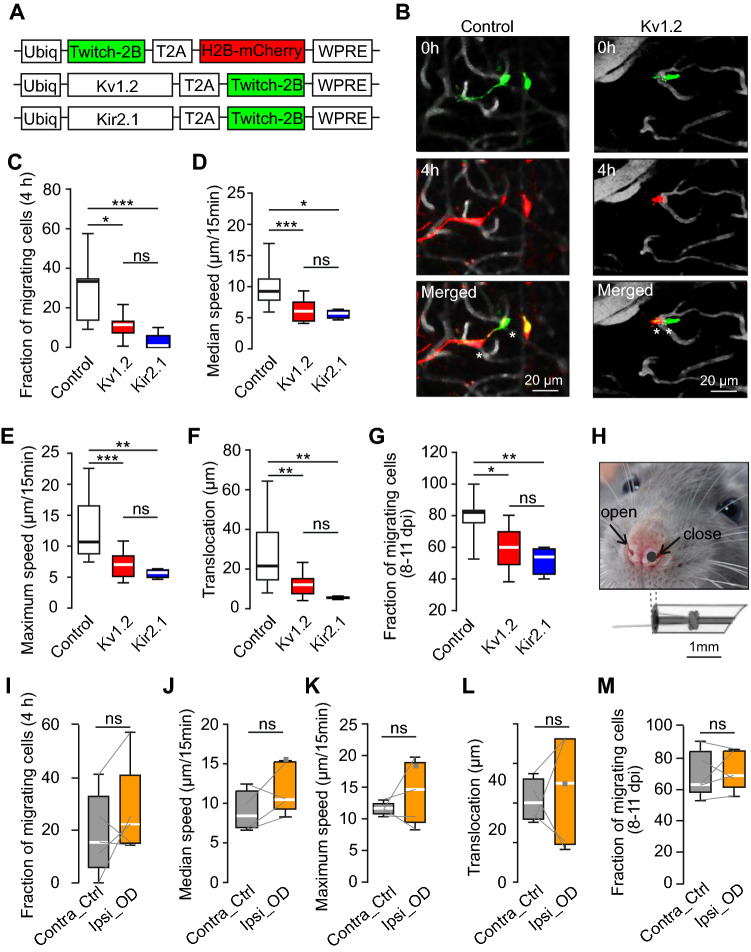
Migration properties of abJGCs. (**A**) Schematics of lentiviral constructs. (**B**) Pseudocolor images showing migrating control (left) and Kv1.2-expressing (right) abJGCs at different relative times (timestamps). Blood vessels are shown in gray, asterisks highlight the positions of migrating cells. Data shown in (**B**–**F**) and (**I**–**L**) are obtained at 8 dpi. (**C**) Box plot showing the median (per mouse) fractions of abJGCs, migrating during 4-h-long recordings in control, Kv1.2, and Kir2.1 groups (n = 11, 10 and 6 mice, respectively). (**D**–**F**) Box plots showing (per cell) the median (**D**) or the maximum (**E**) migration speed and the translocation distance in 4 h (**F**); n = 29/13, 13/9, 4/6 cells/mice for control, Kv1.2 and Kir2.1 groups, respectively. Three mice had no migrating Kir2.1 cells. (**G**) Box plot showing the median (per mouse) fractions of abJGCs, migrating between 8 and 11 dpi; n = 8, 10, 4 mice for control, Kv1.2, and Kir2.1 groups, respectively. (**H**) Image illustrating the nostril occlusion. (**I**–**M**) Box plots showing median fractions of abJGCs migrating in 4 h (**I**), the median (**J**) and maximum (**K**) migration speed, the translocation distance in 4 h (**L**), and the fraction of cells, migrating between 8 and 11 dpi (**M**), in contralateral control and ipsilateral odor-deprived hemibulbs (n = 16/5 and 19/5 cells/mice) for control and odor-deprived groups, respectively. Data obtained from single neurons were averaged per hemibulb. Here and below grey lines connect control and odor-deprived hemibulbs of the same mice. Note that there were no migrating cells during 4 h of recording on dpi 8 in one control hemibulb). Here and below *P < 0.05, **P < 0.01, ***P < 0.001, ns = not significant. All exact P values are listed in Supplementary Table S4

**Fig. 2:**
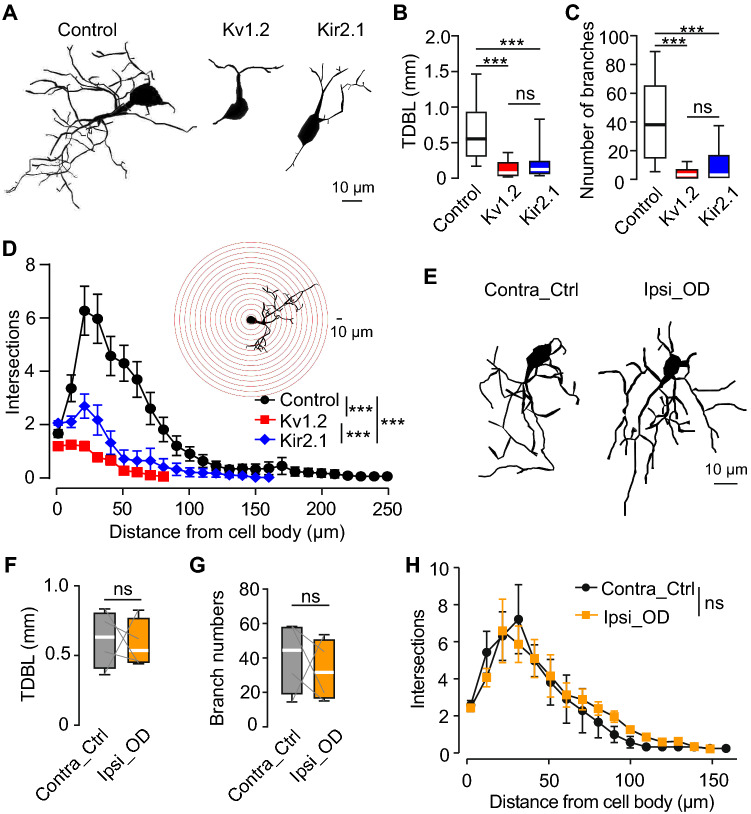
Morphology of abJGCs. (**A**) Sample reconstructions of in vivo abJGCs (20 dpi) belonging to control, Kv1.2, and Kir2.1 groups. (**B**, **C**) Box plots showing (per cell) the total dendritic branch length (TDBL, (**B**)) and the number of dendritic branches (**C**) of abJGCs, imaged in vivo at 20 dpi; n = 33/7, 36/6, and 46/6 cells/mice for control, Kv1.2, and Kir2.1 groups, respectively. (**D**) Sholl analysis, showing the number of intersections of centered Sholl spheres (10 µm step size, illustrated in the inset) with the dendritic trees of abJGCs, belonging to control, Kv1.2 and Kir2.1 groups. n = 33/7, 36/6, 46/6 cells/mice for control, Kv1.2 and Kir2.1 groups, respectively. (**E**) Sample reconstructions of in vivo abJGCs (20 dpi) from the contralateral control and ipsilateral odor-deprived hemibulbs. (**F**, **G**) Box plots illustrating (per cell) the TDBL (**F**) and the number of dendritic branches (**G**) of control and odor-deprived abJGCs. (**H**) Sholl analysis of the dendritic trees of JGCs from control and odor-deprived hemibulbs; n = 9/4 and 20/4 cells/mice for control and odor-deprived groups, respectively. (**D**, **H**) Data are shown as mean ± SEM

Bicistronic lentiviruses encoding a Ca^2+^ indicator Twitch-2B and either Kv1.2 or Kir2.1 (Fig. 1A and Supplementary Fig. S1A) were injected into the RMS to transduce adult-born cells migrating towards the OB [36]. In the control group, mCherry was expressed together with Twitch-2B. The efficiency of virus transduction was confirmed by the level of Twitch-2B fluorescence, the relative amount of the respective mRNA, and the heightened level of Kv1.2 immunofluorescence (Supplementary Fig. S1B-F). In line with previous publications [56, 57], whole-cell patch clamp experiments in acute OB slices assured spiking of both Kv1.2 and Kir2.1 overexpressing cells (days post-injection (dpi) 20; Supplementary Fig. S2A). The detailed analyses of the Kv1.2 group, for which literature data are scarce, revealed that despite the significantly smaller capacitance (mean ± SEM: 6.38 ± 1.52 pF, n = 7 and 13.1 ± 1.74 pF, n = 10 for Kv1.2 and control groups, respectively; t-test, p = 0.01) neither input resistance, nor the AP firing threshold, amplitude, or duration (full width at half maximum (FWHM)) differed significantly between the control and Kv1.2 overexpressing cells (Supplementary Fig. S2B-E). Moreover, the resting membrane potentials were similar between the two groups (control: − 59.58 ± 2.98 mV, n = 9 cells; Kv1.2 group: − 53.79 ± 4.76 mV, n = 7 cells; t-test, P = 0.33). A mild functional signature of the Kv1.2 overexpression was, however, visible when analyzing the afterhyperpolarizations accompanying the AP firing (Fig. 2SF, G). Furthermore, using immunohistochemistry we assessed the expression of the marker of migrating neuroblasts doublecortin and the marker of mature neurons NeuN [8, 58] at dpi 28. As shown in Supplementary Fig. S3, the gross time course of the maturation (i.e., timely downregulation of the doublecortin expression and timely upregulation of the NeuN expression) was similar between control, Kv1.2 and Kir2.1 groups. These data, together with similar and low basal Ca^2+^ levels in control, Kv1.2 and Kir2.1 groups (Fig. S9A), indicate that overexpression of Kv1.2 or Kir2.1 channels does not induce nonspecific toxic effects.

The developmental history of abJGCs after their arrival in the glomerular layer of the bulb was monitored longitudinally through a chronic cranial window from 8 till 45 dpi using in vivo two-photon imaging. To analyze the migration of abJGCs, we measured their positions every 15 min during a 4-h-long imaging session (Fig. 1B). Compared to Twitch-2B/mCherry-expressing control group, the expression of neither Kv1.2 nor Kir2.1 affected the apparent rate of adult-born cells’ arrival in the glomerular layer of the bulb (Supplementary Fig. S4), likely because of the downregulation of potassium channel expression in migration neuroblasts [59]. However, it severely impaired cell migration at 8 dpi in the glomerular layer (Fig. 1C). The median (per mouse) fraction of migrating cells was 33.3 ± 21.7% in the control group but decreased significantly in the Kv1.2 and Kir2.1 groups (12.1 ± 6.6% and 0 ± 5.9%, respectively). Moreover, the median and maximum speed of migrating cells (Fig. 1D, E) as well as the cumulative translocation in 4 h (Fig. 1F) decreased significantly in the Kv1.2 and Kir2.1 groups compared to the control group. Since abJGCs have a saltatory migration pattern [36], some of them might pause during the 4-h-long recording period. Therefore, we also evaluated the overall motility of abJGCs, defining the cells, which changed their positions between 8 and 11 dpi, as migrating. In the control group, 82.1 ± 8.8% of cells migrated at 8–11 dpi (see also [36]). This fraction was significantly lower in the Kv1.2 and Kir2.1 groups (Fig. 1G; 60 ± 21.8% and 54 ± 17% of cells, respectively).

The observed impairment of migration in Kv1.2 and Kir2.1 groups was probably chronic, as it persisted at 14 dpi (Supplementary Fig. S5). By this time the lateral migration of control cells is known to slow down [36]. Likely therefore there was no difference in the fraction of migrating cells as well as the median and maximum migrating speed between the control and Kv1.2 groups (Supplementary Fig. S5A-C). Yet, the significantly shorter net translocation distance in the Kv1.2 group (Supplementary Fig. S5D) suggested that cell motility was impaired. At 14 dpi Kir2.1 expressing cells were not migrating at all (Supplementary Fig. S5A). This finding is consistent with the somewhat stronger impact of Kir2.1 on cell migration compared to Kv1.2, visible throughout the data (Fig. 1C–G).

Next, we explored whether modulation of sensory input impacts the migration of abJGCs. Because odors are inhaled through the nostrils into two segregated nasal passages [60], we occluded one nostril and thus also the ipsilateral hemibulb, leaving the other nostril open. Nostril occlusion (Fig. 1H) starting at 5 dpi completely blocked odor-evoked Ca^2+^ transients in abJGCs residing in the ipsilateral hemibulb (measured at 20 dpi), while leaving the cells in the control hemibulb unaffected (compare Supplementary Fig. S6 to Fig. 4). Surprisingly, odor deprivation did not affect any of the migration parameters (Fig. 1I–M). All parameters were similar for cells residing in the contralateral control and the ipsilateral odor-deprived hemibulbs. Moreover, odor deprivation did not affect the translocation distance in 12 h at 6.5–14.5 dpi, repeatedly measured by us previously (see Fig. S4 in ref. [36]). Together, these data suggest that endogenous but not sensory-driven activity is critical for the lateral migration of abJGCs during the pre-integration phase.

### Endogenous but not sensory-driven activity controls morphogenesis of adult-born JGCs

Immature adult-born cells in the RMS maintain a spindle shape morphology to enable fast migration but grow complex dendrites shortly after reaching the glomerular layer [19, 30, 61]. Surprisingly, even at 20 dpi abJGCs in Kv1.2 and Kir2.1 groups displayed remarkably retarded morphology with significantly shorter total dendritic branch length (TDBL), fewer dendritic branches and intersections (Fig. 2A–D). Control cells had a median (per cell) TDBL of 553 ± 630 µm and a branch number of 38 ± 50. These values, however, dropped significantly to 75 ± 197 µm/122 ± 187 µm (TDBL) and 3 ± 6/3 ± 16 (branch number) in Kv1.2 and Kir2.1 groups, respectively (Fig. 2B, C). Similar differences were observed in the fixed tissue by means of immunohistochemistry (Supplementary Fig. S7). In 50-µm-thick slices, however, the length and complexity of all cells were somewhat reduced. The observed morphological retardation was specific for functional Kv1.2/Kir2.1 channels, as expressing the non-conducting dominant-negative mutant of the Kir2.1 channel (Kir2.1mut) [62] within the adult-born cells did not inhibit their morphogenesis (Supplementary Fig. S8). Besides, no changes in morphology we observed in abJGCs, residing in the control and odor-deprived hemibulbs (Fig. 2E–H). Together, these results suggest that endogenous but not sensory-driven activity is essential for the proper morphological development of abJGCs.

### Altered endogenous activity reduced the survival rate of adult-born JGCs

**Fig. 3:**
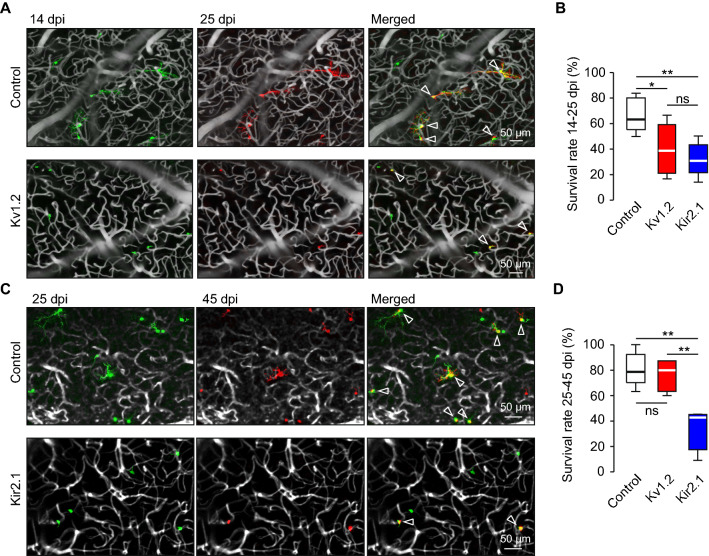
Kv1.2 or Kir2.1 overexpression reduced the survival of abJGCs. (**A**) Sample maximum intensity projection (MIP) images showing abJGCs at 14 (colored green) and 25 dpi (colored red) in control (40–80 µm below the dura) and Kv1.2 (20–50 µm below the dura) groups. All images show the central part of the FOV. Here and below arrowheads point to stable surviving cells. Here and below the blood vessels are colored grey. (**B**) Box plot showing the survival rates of abJGCs between 14–25 dpi in control, Kv1.2 and Kir2.1 groups; n = 40/5, 81/8 and 52/5 cells/mice for control, Kv1.2 and Kir2.1 groups, respectively. (**C**) Sample MIP images showing abJGCs at 25 (colored green) and 45 dpi (colored red) in control (13–80 µm below the dura) and Kir2.1 (33–65 µm below the dura) groups. (**D**) Box plot showing the survival rates of abJGCs between 25–45 dpi in control, Kv1.2, and Kir2.1 groups; n = 82/5, 23/5, and 49/4 cells/mice for control, Kv1.2 and Kir2.1 groups, respectively

Next, we analyzed the survival of abJGCs. Because the rapid migration of the cells at the beginning of the pre-integration phase [36] makes their precise identification difficult, we started experiments at 14 dpi. Utilizing a priori knowledge about the average speed of cell migration [36], we set a safety margin between the analyzed cells and the edge of the field of view (FOV) to ensure that cells, disappearing from the FOV, did not simply move out. Cells, which stayed at the same position during the recording period, were considered stable surviving cells (Fig. 3A, C). The expression of either Kv1.2 or Kir2.1 significantly reduced the survival rate of abJGCs between 14 and 25 dpi (Fig. 3B). Under these conditions, the mean (per mouse) survival rate was 66 ± 5.6% in control, 40 ± 6.4% in Kv1.2, and 32 ± 5.8% in the Kir2.1 group. Between 25 and 45 dpi, survival was still inhibited significantly in the Kir2.1 group (35.6 ± 8.9% vs. 82 ± 5.6% in control) but not in the Kv1.2 group (76.3 ± 5.6%) (Fig. 3D), likely because the activity of cells increased during development, thus reducing the inhibitory effect of surplus Kv1.2 channels. These data suggest that reduced endogenous activity inhibits the survival of abJGCs during the pre-integration phase, with somewhat stronger effects observed in the Kir2.1 compared to the Kv1.2 group.

### Weaker integration of adult-born JGCs with altered endogenous activity into the existent neural circuitry

**Fig. 4:**
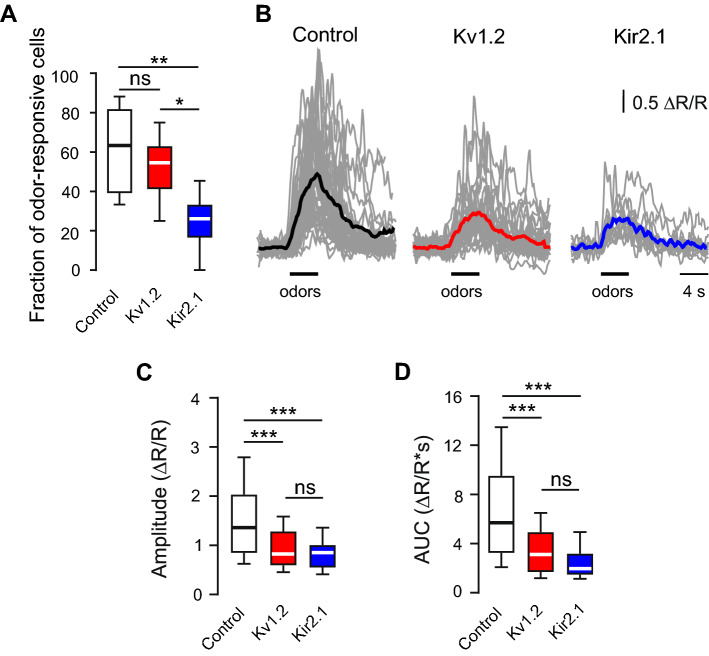
Kv1.2 or Kir2.1 overexpression inhibited odor-evoked Ca^2+^ transients in abJGCs. (**A**) Box plot showing the fractions of odor-responsive abJGCs in control, Kv1.2 and Kir2.1 groups (20 dpi); n = 78/6, 59/7 and 63/6 cells/mice for control, Kv1.2 and Kir2.1 groups, respectively. (**B**) Sample traces (gray) showing the odor-evoked Ca^2+^ transients of abJGCs at 20 dpi. Colored bold traces are the respective means. (**C**, **D**) Box plots summarizing the median (per cell) amplitudes (**C**) and areas under the curve (AUC, (**D**)). (**B**–**D**) n = 82/7, 53/7, and 17/5 cells/mice for control, Kv1.2, and Kir2.1 groups, respectively

Next, we explored whether adult-born cells in Kv1.2/Kir2.1 groups were able to integrate into the existent neural network. We used the fraction of odor-responsive abJGCs, the amplitude and the area under the curve (AUC) of the odor-evoked Ca^2+^ signals (measured at dpi 20) as functional readouts of the strength of their network integration. The overexpression of either Kv1.2 or Kir2.1 diminished the odor responsiveness of abJGCs (Fig. 4A). The mean (per mouse) fraction of odor-responsive cells was 61.5 ± 8.6% in the control group, decreasing slightly (to 51 ± 6.1%) in the Kv1.2 and significantly (to 24.8 ± 6%) in the Kir2.1 groups. The amplitudes and AUCs of odor-evoked Ca^2+^ transients in the Kv1.2 and Kir2.1 groups were significantly smaller than in the control group (Fig. 4B–D). The median (per cell) amplitude of odor-evoked Ca^2+^ transients reached 1.36 ± 1.19 ΔR/R in the control group, 0.83 ± 0.69 ΔR/R in the Kv1.2 group, and 0.85 ± 0.46 ΔR/R in the Kir2.1 group (Fig. 4C). The median (per cell) AUC of odor-evoked Ca^2+^ transients was 5.7 ± 6.3 ΔR/R*s in the control, 3.1 ± 3.2 ΔR/R*s in the Kv1.2 and 2.0 ± 1.7 ΔR/R*s in the Kir2.1 group (Fig. 4D). Thus, abJGCs with altered endogenous activity have difficulties to integrate into the existent OB circuitry.

### Spontaneous Ca^2+^ transients in adult-born JGCs were dampened by overexpression of Kv1.2 or Kir2.1 but maintained under odor deprivation

**Fig. 5:**
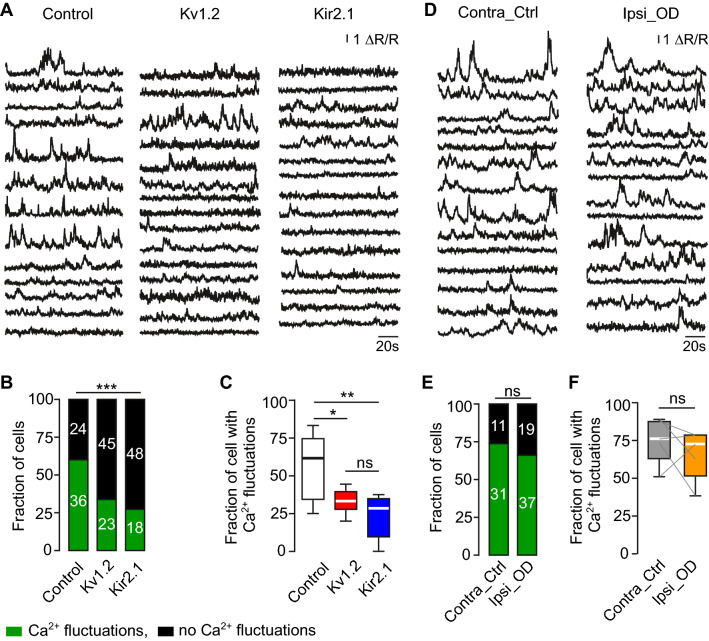
Kv1.2 or Kir2.1 overexpression perturbed the pattern of spontaneous Ca^2+^ transients in abJGCs while odor deprivation did not. (**A**) Sample traces illustrating spontaneous Ca^2+^ transients measured in awake mice from abJGCs in control, Kv1.2, and Kir2.1 groups. (**B**) Stacked bars showing the fractions of cells with and without fluctuations in [Ca^2+^]_i_. Here and in (**E**) the numbers of cells in each category are given within the bars. (**C**) Box plot illustrating the median (per mouse) fractions of abJGCs with fluctuations in [Ca^2+^]_i_. (**B**, **C**) n = 60/8, 68/7, and 66/5 cells/mice for control, Kv1.2, and Kir2.1 groups, respectively. (**D**) Sample traces illustrating spontaneous Ca^2+^ transients of abJGCs from contralateral control and ipsilateral odor-deprived hemibulbs. (**E**) Stacked bars showing the fractions of cells with and without fluctuations in [Ca^2+^]_i_ in control and odor-deprived groups. (**F**) Box plot illustrating the median (per mouse) fractions of abJGCs with fluctuations in [Ca^2+^]_i_ in control and odor-deprived groups. (**E**, **F**) n = 34/4 and 47/4 cells/mice for contralateral control and ipsilateral odor-deprived groups, respectively

To extract features of the endogenous activity crucial for migration, morphogenesis, survival, and integration of abJGCs, we compared (at 12 dpi) the patterns of endogenous Ca^2+^ signaling recorded in control, Kv1.2, Kir2.1 groups and in two groups from odor-deprived mice (cells, residing in odor-deprived and control hemibulbs). In abJGCs, the overexpression of Kv1.2 or Kir2.1 did not affect the basal Twitch-2B ratios (reflecting basal levels of the intracellular free Ca^2+^ concentration ([Ca^2+^]_i_)), maximum Twitch-2B ratios, and normalized AUCs (Supplementary Fig. S9A-C). However, it profoundly reduced the fraction of cells with spontaneous fluctuations in [Ca^2+^]_i_ (Fig. 5A). Using the Gaussian mixture model [44], 36 (out of 60 cells) with fluctuations in [Ca^2+^]_i_ were identified in the control group, whereas in the Kv1.2 group only 23 out of 68 cells and in the Kir2.1 group only 18 out of 66 cells showed such fluctuations (Fig. 5B). The mean (per mouse) fraction of cells with fluctuations in [Ca^2+^]_i_ amounted to 57.5 ± 7.8% in the control, 33.5 ± 3.1% in the Kv1.2, and 23.5 ± 6.7% in the Kir2.1 group (Fig. 5C). Thus, the overexpression of Kv1.2 or Kir2.1 significantly dampens spontaneous fluctuations in [Ca^2+^]_i_.

In contrast, no significant changes in the pattern of endogenous activity were found in odor-deprived cells (Fig. 5D–F). The mean fraction of cells with fluctuations in [Ca^2+^]_i_ per mouse was 74.2 ± 6.7% in the contralateral control and 65.4 ± 7.5% in the ipsilateral odor-deprived group (Fig. 5F). Both values were similar to those measured in the control group. Besides, there was no significant difference in the basal and maximum Twitch-2B ratios as well as AUCs between the contralateral control and the ipsilateral odor-deprived groups (Supplementary Fig. S9D–F). We noticed, however, that in both the contralateral control and the ipsilateral odor-deprived groups the basal Twitch-2B ratios and AUCs were slightly but significantly smaller than the respective values measured in mice without nostril occlusion (i.e., control, Kv1.2, and Kir2.1 groups; Kruskal–Wallis test: for basal Twitch-2B ratios, *P* = 5.1 × 10^–5^; for AUCs, *P* = 5.2 × 10^–5^), likely reflecting the blockade of the sniff-driven mechanosensation [63]. As migration and morphology were not affected in odor-deprived mice, these data suggest that these parameters were not important for the proper development of abJGCs. Together, these data identify the patterned endogenous activity consisting of fluctuations in [Ca^2+^]_i_ as a key parameter likely governing the migration, morphogenesis and survival of abJGCs.

### The role of pCREB in regulation of early neuronal development

**Fig. 6:**
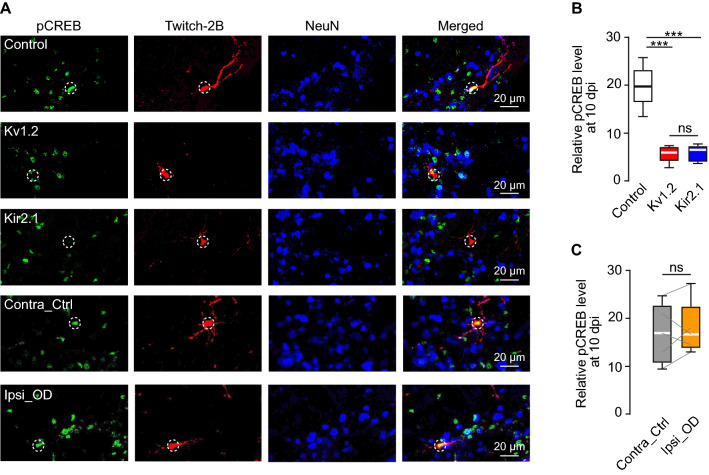
Kv1.2 or Kir2.1 overexpression downregulated the pCREB signaling pathway. (**A**) Sample MIP images (20 µm depth) showing pCREB-, Twitch-2B- and NeuN-positive cells in the glomerular layer of the OB slices from control, Kv1.2, Kir2.1, Contra_Ctrl, and Ipsi_OD groups at 10 dpi. Twitch-2B labels the abJGCs whereas NeuN labels the mature neurons. Circles highlight the locations of abJGCs. (**B**, **C**) Box plots illustrating the median (per mouse) relative pCREB levels of abJGCs in different experimental groups, as indicated, at 10 dpi. n = 152/6, 230/6, 177/ 5, 129/5, and 94/5 cells/mice for control, Kv1.2, Kir2.1, Contra_Ctrl, and Ipsi_OD groups, respectively

Phosphorylation of the cAMP response element-binding protein (CREB) at the Ser133 residue is known to play an important role in the differentiation and survival of adult-born cells [11, 64]. Typically, pCREB is abundant in developing and low in mature neurons [65]. We, therefore, measured the levels of pCREB in our 5 experimental groups (control, Kv1.2, Kir2.1, contralateral control, ipsilateral odor-deprived). Adult-born JGCs in the Kv1.2 and Kir2.1 groups showed a significant decrease in the level of pCREB during the pre-integration phase (Fig. 6). At 10 dpi, the mean (per mouse) relative pCREB level (i.e., normalized to the mean fluorescence intensity of pCREB of all surrounding mature NeuN-positive cells) of abJGCs was 19.7 ± 1.7 in control but decreased significantly to 5.6 ± 0.7 in the Kv1.2 and 5.8 ± 0.8 in the Kir2.1 group (Fig. 6B). The pCREB levels in odor-deprived mice (16.8 ± 2.8 in the contralateral control and 17.9 ± 2.5 in the ipsilateral odor-deprived group) were similar to control and significantly different from Kv1.2/Kir2.1 groups (Fig. 6C). As the pCREB level in adult-born cells gradually decreases during their maturation [65], at 28 dpi the respective values were 4.6 ± 0.5 for the control, 1.5 ± 0.2 for the Kv1.2, and 1.4 ± 0.2 for the Kir2.1 group (Tukey's multiple comparison test: control vs. Kv1.2: *P* = 1 × 10^–5^; control vs. Kir2.1: *P* = 7 × 10^–5^; Kv1.2 vs. Kir2.1: *P* = 0.95; n = 110/5, 143/5, and 161/5 cells/mice, respectively). Thus, in abJGCs with dampened endogenous activity, the pCREB signaling pathway is strongly inhibited throughout the pre-integration phase.

### Transcriptomic analyses of pathways linked to pCREB signaling

**Fig. 7:**
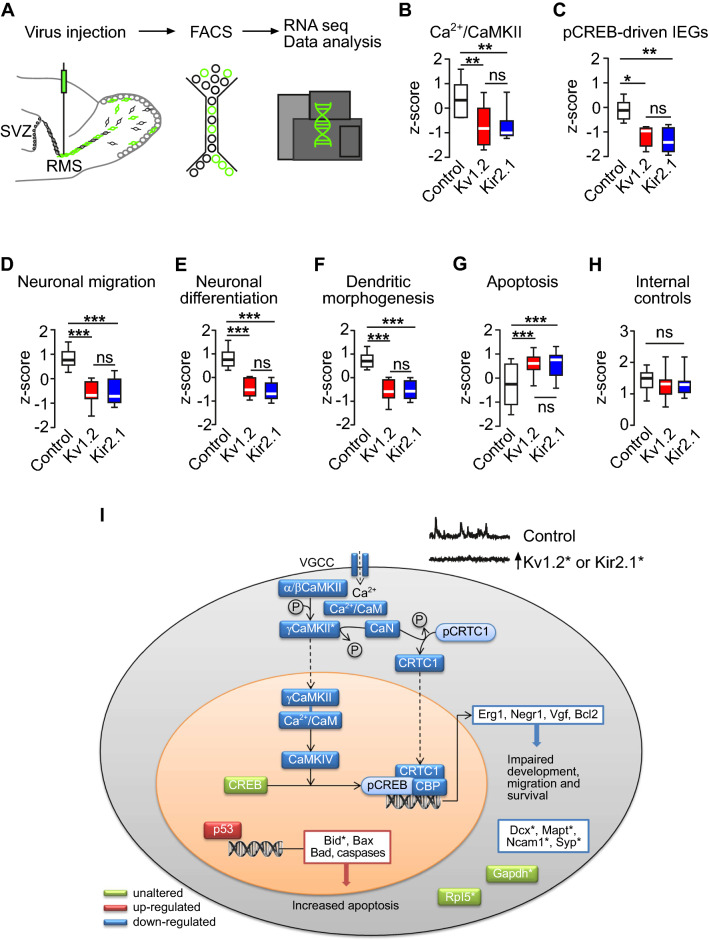
Transcriptome analyses of adult-born cells. (**A**) Schematic illustration of the workflow used for RNAseq. (**B**–**H**) Box plots showing the expression levels of genes in the modules of Ca^2+^/CaMKII (**B**), pCREB-driven IEGs (**C**), neuronal migration GO:0001764 (**D**), neuronal differentiation GO:0045664 (**E**), dendritic morphogenesis GO:0048813 (**F**), apoptotic process GO:0006915 (**G**), and 9 housekeeping genes serving as internal controls (**H**). All transcripts included in the box plots are listed in Supplementary Table S1. n = 2 biological replicates (12 mice in total, 2 mice per replicate per group). (**I**) The schematic summary of the genes/pathways involved. See the discussion for further details. Asterisks indicate genes, the expression of which was verified by qPCR

To investigate the molecular pathways that might link the dysregulation of abJGCs’ Ca^2+^ signaling to the reduction in pCREB, we isolated adult-born cells belonging to the control, Kv1.2, and Kir2.1 groups using fluorescence-activated cell sorting (FACS) and determined their transcriptomic profiles using next-generation sequencing (Fig. 7). For a subset of genes (i.e., one representative member of pathways analyzed in Fig. 7), the data obtained were further validated by quantitative real-time PCR (Supplementary Fig. S10). Because the FACS-sorted cells, isolated from the OB, also contained adult-born GCs, we ensured that adult-born GCs recapitulated the impairment in morphology and CREB phosphorylation, described above for abJGCs (Supplementary Fig. S11).

As expected, the data revealed a significant upregulation of the *Kcna2* and *Kcnj2* mRNAs in the Kv1.2 and Kir2.1 groups (Supplementary Fig. S1E, F), as well as a stable expression pattern of housekeeping genes (Fig. 7H, Supplementary Fig. S10G). Interestingly, the cells in the Kv1.2 and Kir2.1 groups showed a significantly lower expression of genes participating in the transport of the Ca^2+^/Calmodulin complex (Ca^2+^/CaM) into the nucleus, such as several isoforms of the Ca^2+^/CaM-dependent protein kinase II (CaMKII) (Fig. 7B; for details, see Supplementary, Table S1). Activation of this shuttle pathway is mediated by Ca^2+^ influx through either voltage-gated L-type Ca^2+^ channels (VGCC; Fig. 7I) or NMDA receptors (Supplementary Fig. S12A). The final step in the cascade involves the activation of the CaMKIV, which is responsible for pCREB phosphorylation. Transcripts, encoding the L-type Ca^2+^ channels, NMDA receptors, and CaMKIV, were also downregulated in the Kv1.2 and Kir2.1 groups (Fig. 7B, I and Supplementary Fig. S12A). Moreover, additional molecules, required to fully activate the transcription complex of pCREB, like the CREB-regulated transcription coactivator 1 (CRTC1) and calcineurin (CaN), which dephosphorylates CRTC1 to allow its translocation into the nucleus and binding to the transcription unit, were also downregulated (Fig. 7I). Next, we investigated further molecules, which act as coactivators or repressors within the transcription unit of pCREB, e.g., LIM domain only 4 (LMO4), DRE-Antagonist Modulator (DREAM), Calcium-Responsive Transactivator (CREST) [66, 67]. The expression profile of these molecules was consistent with the decreased transcriptional activity of pCREB (Supplementary Table S2). Notably, the other major pathway leading to the phosphorylation of CREB, namely the MAPK/ERK pathway, remained unchanged (Supplementary Fig. S12B).

In accordance with the above findings, we observed a decreased expression of pCREB-driven immediate early genes (e.g., early growth response 1 (*Egr1*), neuronal growth regulator 1 (*Negr1*), etc.; Fig. 7C, I) and downregulation of pathways responsible for neuronal migration (GO:0001764), differentiation (GO:0045664) as well as dendritic morphogenesis (GO:0048813) in the Kv1.2 and Kir2.1 groups (Fig. 7D–F, I; Supplementary Fig. S10, Table S1, S5). These results are consistent with the in vivo functional properties of abJGCs overexpressing Kv1.2 or Kir2.1 channels. In line with the reduced survival of abJGCs in the Kv1.2 and Kir2.1 groups (Fig. 3), the transcripts involved in the apoptotic process (GO:0006915) were upregulated, while the anti-apoptotic genes, like the apoptosis regulator *Bcl2*, were downregulated (Fig. 7G, I; Supplementary Fig. S10, Table S1). Together, these data identify a defined signaling pathway, connecting the specific pattern of endogenous neuronal activity to downstream signaling cascades regulating migration, differentiation, morphogenesis, and survival of adult-born neurons.

## Discussion

This study focused on the pre-integration phase [36], to examine the role of endogenous as well as sensory-driven activity in the key aspects of abJGCs’ development including migration, differentiation, morphogenesis, and survival, and to identify the possible molecular mechanisms involved. Against the widespread belief in the field (see Introduction), sensory-driven activity had little impact on the lateral migration and morphogenesis of abJGCs, despite their vivid odor responsiveness already at dpi 9 [19]. Instead, reducing the abJGCs’ endogenous activity (via cell-specific overexpression of Kv1.2/Kir2.1 K^+^ channels) dampened the cytosolic fluctuations in [Ca^2+^]_i_, expression (and likely also activation) of the voltage-gated and NMDA receptor channels as well as phosphorylation of CREB (Fig. 7, Supplementary Fig. S12) and dramatically retarded the migration, differentiation, morphogenesis, and survival of abJGCs. Our transcriptomic data identified the candidate genes, which might relay this Ca^2+^/pCREB signal further to govern the integration of abJGCs into existing neural circuitry.

Several lines of evidence suggest that the effects observed are caused by downregulation of the endogenous activity, intracellular Ca^2+^ signaling (visualized by decreased fluctuations in [Ca^2+^]_i_) and Ca^2+^/pCREB-regulated pathway (Fig. 7I) rather than nonspecific toxic effects of overexpressed proteins: (i) under our experimental conditions the protein overexpression was rather mild (Supplementary Fig. S1); (ii) basal Ca^2+^ levels in control, Kv1.2 and Kir2.1 groups were similar and low (Fig. S9A); (iii) electrophysiological properties of Kv1.2 cells did not show any overt abnormalities (Supplementary Fig. S2); (iv) the disappearance of immature cell marker doublecortin and the appearance of the marker of mature neurons NeuN happened simultaneously in control, Kv1.2 and Kir2.1 groups (Supplementary Fig. S3); (iv) out of 11,611 genes detected by RNAseq, only 7.62% showed reliable twofold changes between the control and Kv/Kir groups. The remaining 92.38% of all detected genes showed similar expression levels in all three groups. Finally (v) while the CaMKIV/pCREB pathway was strongly downregulated (Fig. 7I) the other major pathway leading to phosphorylation of CREB, namely the MAPK/ERK pathway, remained unchanged (Supplementary Fig. S12B).

Regarding morphogenesis, our data are consistent with that of A. Mizrahi [32] but fit less well with his later data, showing almost doubling of the TDBL in cells, exposed to an odor-enriched environment [21]. Besides, our data align with that obtained in adult-born GCs, whose migration and morphology were normal in anosmic mice lacking any electrical activity in the olfactory epithelium [30], as was the adult-born GCs morphology in young adult mice after 5 weeks of naris occlusion [68]. On the other hand, 30–40 days of naris occlusion starting at postnatal days 4–5 caused a significant reduction of TDBL of GCs [69]. Moreover, naris occlusion also impeded the migration of neuroblasts along the RMS and the tenascin-R-dependent radial migration of neuroblasts from the RMS to the olfactory bulb [16, 17]. Longitudinal monitoring of cells’ basal and manipulation-induced activity (e.g., by means of Ca^2+^ imaging [37]), as well as a clear separation between early postnatal and adult neurogenesis, might help to shed more light on the above discrepancies. The literature data are, however, consistent regarding the negative impact of naris occlusion on the survival and synapse/spine density of adult-born OB interneurons [27–30, 68, 70].

Here, we show that the lateral migration, differentiation, morphogenesis, and survival of abJGCs are significantly impaired in cells with reduced endogenous activity, which we directly visualized in vivo by means of Ca^2+^ imaging (Fig. 5A). The endogenous activity is present during all stages of the adult OB neurogenesis, i.e., in neural progenitor cells in the SVZ [71], in neuroblasts in the RMS [12, 72] and adult-born cells in the subependymal [73] as well as glomerular [38] layers of the OB. Still, its reduction by means similar to the ones used in our study had little impact on tangential and radial migration of adult- and postnatally-born GCs as well as positioning and survival of postnatally-born JGCs [34, 35]. Together, these findings further emphasize the differences in the maturation programs of GCs and JGCs as well as the adult- and postnatally-born OB interneurons and show that data described in this study cannot be envisaged from the literature.

The importance of ongoing fluctuations in [Ca^2+^]_i_ for the abJGC maturation process can be explained by the interplay of the CaMKII- and the CRTC1-dependent pathways. Both pathways are triggered by the activation of L-type voltage-gated Ca^2+^ channels (and NMDA receptors) and require the Ca^2+^/calmodulin (CaM) complex [66, 74, 75]. While the CaMKII-dependent pathway leads to the γCaMKII-mediated shuttling of Ca^2+^/CaM to the nucleus and the subsequent phosphorylation of CREB [66, 74, 76], the calcineurin (CaN)-mediated dephosphorylation of CRTC1 supports CRTC1 translocation to the nucleus, where it is required for the pCREB-driven gene expression [77, 78]. One such pCREB/CRTC1-dependent target gene is salt-inducible kinase 1 (SIK1), which phosphorylates CRTC1, triggering its export from the nucleus and thus arresting pCREB/CRTC1-mediated transcription [77]. Via this mechanism long-lasting steady-state elevations of [Ca^2+^]_i_ weaken rather than potentiate the pCREB-mediated gene expression. In the Kv1.2 and Kir2.1 groups analyzed in our study, we also observed the downregulation of transcripts involved in these important pathways (Fig. 7), thus further impairing the signal transduction from the cell membrane to the nucleus. Interestingly, the molecular pathways described above are similar to the ones (i) operating during neonatal neurogenesis [34, 35, 57, 77, 79–82] or (ii) governing activity-dependent synaptic plasticity and memory acquisition/retrieval in the adult brain [66, 83, 84].

The decreased expression of anti-apoptotic (Bcl2) and the increased expression of pro-apoptotic (Bid, Bax, Bad, caspases) genes, revealed by our study, is consistent with our longitudinal imaging data, documenting decreased survival of adult-born cells. The apoptosis can be either fostered by reduced neuronal activity/ fluctuations in [Ca^2+^]_i_ [85, 86] or directly triggered by the enhanced transmembrane K^+^ efflux (reviewed in ref. [87]).

Taken together, our data show that despite developing in a rich sensory environment and being able to respond to sensory stimuli from early on [19, 20, 88], adult-born neurons strongly rely on cell-intrinsic activity for their morphogenesis and positioning within the glomerular layer. By providing several missing links, our data revealed signaling pathways connecting the intermittent neuronal activity/ongoing fluctuations in [Ca^2+^]_i_ on one side and the increased transmembrane K^+^ efflux on the other side to neuronal migration, maturation, and survival.

### Supplementary Information

Below is the link to the electronic supplementary material.

Supplementary file1 (PDF 4131 KB)

Supplementary file2 (XLSX 48 KB)

## Data Availability

The authors declare that all data supporting the findings of this study are available within the paper. The RNA sequencing data are uploaded to *Gene Expression Omnibus* (*GEO number: GSE168349*) database repository before publication. All data, code and materials in this study are also available upon reasonable request from corresponding author Olga Garaschuk at olga.garaschuk@uni-tuebingen.de.
