# Supplementary material for: Endogenous but not sensory-driven activity controls migration, morphogenesis and survival of adult-born juxtaglomerular neurons in the mouse olfactory bulb
Source: Cell Mol Life Sci. 2023 Mar 18;80(4):98. doi: 10.1007/s00018-023-04753-4 (PMC10023654; doi:10.1007/s00018-023-04753-4)
Supplement: Supplementary file 1 — Supplementary file1 (PDF 4131 KB) [file 18_2023_4753_MOESM1_ESM.pdf]

Supplementary Materials

for

Endogenous but not sensory-driven activity controls migration, morphogenesis and survival of adult-born neurons in the mouse olfactory bulb

Kaizhen Li[1,7], Katherine Figarella[1,8], Xin Su[1,8], Yury Kovalchuk[1], Jessika Gorzolka[1], Jonas J. Neher[2,3], Nima Mojtahedi[1], Nicolas Casadei[4,5], Ulrike B. S. Hedrich[6], Olga Garaschuk[1,9]

[1], Department of Neurophysiology, Institute of Physiology, University of Tübingen, Tübingen, Germany

[2], German Center for Neurodegenerative Diseases (DZNE), Tübingen, Germany

[3], Department of Cellular Neurology, Hertie Institute for Clinical Brain Research, University of Tübingen, Tübingen, Germany

[4], Institute of Medical Genetics and Applied Genomics, University of Tübingen, Tübingen, Germany

[5], NGS Competence Center Tübingen, Tübingen, Germany

[6], Department of Neurology and Epileptology, Hertie Institute for Clinical Brain Research, University of Tübingen, Tübingen, Germany

[7], Current address: Department of Physiology, University of Bern, Bern, Switzerland

This PDF file includes:

Supplementary Figure Legends

Supplementary Figures S1 to S12

Supplementary Tables S1 to S4

Supplementary Figure S1. Bicistronic lentiviral vectors induced simultaneous expression of Kv1.2/Kir2.1 and Twitch-2B. (A) Sample images showing the transient expression of the lentiviral vectors in HEK-293T cells. (B) Sample MIP immunofluorescence images (20 μm depth) showing the expression of Kv1.2 in abJGCs at 10 dpi in control (upper panel) and Kv1.2 (lower panel) groups. Arrowheads highlight the location of abJGCs. (C) Cumulative distributions of the relative Kv1.2 expression levels in control and Kv1.2-expressing abJGCs. (D) Box plot showing the median fractions (per mouse) of abJGCs with a ring-shape Kv1.2 expression pattern, likely reflecting enhanced somatic targeting of the protein. (C, D) n=96/6 and 155/6 cells/mice for control and Kv1.2 groups, respectively. (E, F) Bar graphs showing the mean relative expression levels of mRNA encoding for the potassium voltage-gated channel subfamily A member 2 (*Kcna2*, encoding Kv1.2. (E)) and the potassium inwardly rectifying channel subfamily J member 2 (*Kcnj2*, encoding Kir2.1. (F)) as determined by qPCR analyses of FACS-sorted adult-born cells (see SI Materials and Methods). Data are shown as median ± IQR. **P*<0.05, ****P*<0.001, ns=not significant.

Supplementary Figure S2. Electrical properties of Kv1.2/Kir2.1-overexpressing abJGCs. (A) Trains of action potentials, evoked by step-like depolarizing injection currents of +20 pA (schematically depicted below the traces) in control (upper panel), Kv1.2- (middle panel) and Kir2.1-overexpressing abJGCs (dpi 20). The double arrow in the middle panel marks the so called distance to baseline, analyzed in F; the boxed area is expanded in the inset to better visualize the afterhyperpolarization, analyzed in G. (B-G) Box plots comparing the input resistance measured in voltage-clamp mode (B; control: 0.9 ± 1.6 GΩ, n=20 cells; Kv1.2-overexpression: 1.5 ± 2.5 GΩ, n=15 cells); the AP firing threshold (C; control: -34.2 ± 6.0 mV, n = 9 cells; Kv1.2-overexpression: -41.8 ±18.4 mV, n = 7cells); the AP amplitude (D; control: 95.5 ± 45.3 mV, n = 9 cells;

Kv1.2-overexpression: 89.5 ± 42.8 mV, n = 7 cells); the width of APs measured at half maximum (FWHM) (E; control: 0.49 ± 0.32 ms, n = 9 cells; Kv1.2-overexpression: 0.46 ± 0.27 ms, n = 7 cells); the median (per cell) distance from a minimum voltage value measured after individual APs (in bursts of 6 APs induced by 100 ms depolarizing pulses) to the baseline voltage (F; control: 15 ± 1.4 mV, n = 6 APs in 5 cells; Kv1.2-overexpression: 8.7 ± 1.5 mV, n = 6 APs in 5 cells); and the burst afterhyperpolarization, measured as a most negative voltage reached after the burst end relative to the baseline voltage 50-100 ms after the end of current injection (G; control: +2.3 ± 3.4 mV, Kv1.2-overexpression: -2.6 ± 4.1 mV; the same traces as in (F)). *P<0.05, ***P<0.001, ns=not significant.

Supplementary Figure S3. Expression of NeuN and DCX in adult-born JGCs. (A) Sample MIP immunofluorescence images (20 µm depth) showing the adult-born JGCs (Twitch-2B) and NeuN positive cells at 28 dpi. (B) Sample MIP immunofluorescence images (20 µm depth) showing the adult-born JGCs (Twitch-2B) and DCX positive cells at 28 dpi. (C) Box plot showing the fractions (per mouse) of adult-born JGCs, expressing NeuN. n=165/5, 155/5, and 159/5 cells/mice for control, Kv1.2 and Kir2.1 respectively. (D) Box plot showing the fractions (per mouse) of adult-born JGCs, expressing DCX. n=170/5, 171/5, and 180/5 cells/mice for control, Kv1.2 and Kir2.1 respectively. Data are shown as median ± IQR. ns=not significant.

Supplementary Figure S4. Kv1.2 or Kir2.1 overexpression did not influence the arrival of adult-born JGCs in the glomerular layer. Box plots showing the median (per 3D stack volume) density of abJGCs in the OB glomerular layer (i.e., number of abJGCs divided by the respective 3D stack volume) at 8 dpi (A) and 14 dpi (B) in control, Kv1.2 and Kir2.1 groups. (A) n = 57/5, 131/9, and 65/6 cells/mice for control,

Kv1.2, and Kir2.1 groups, respectively. (B) n = 71/5, 89/7, and 60/4 mice/mice for control, Kv1.2, and Kir2.1 groups, respectively. Data are shown as median ± IQR. ns=not significant.

Supplementary Figure S5. Kv1.2 or Kir2.1 overexpression impaired the migration of adult-born JGCs at 14 dpi. (A) Box plot showing the median (per mouse) fractions of abJGCs migrating during 4-hour-long recordings in control, Kv1.2, and Kir2.1 groups. Here and below n=30/10, 5/7, and 0/4 migrating cells/mice for control, Kv1.2 and Kir2.1 groups, respectively. In total, we analyzed 216/10, 86/7, and 60/4 cells/mice for control, Kv1.2, and Kir2.1 groups, respectively. (B-D) Box plots showing (per cell) the median migration speed (B), maximum migration speed (C), and the translocation distance in 4 hours (D) of abJGCs in control and Kv1.2 groups. Data are shown as median ± IQR. *$P<0.05$, ns=not significant.

Supplementary Figure S6. Nostril occlusion blocked odor-evoked responsiveness of adult-born JGCs. (A) Sample odor-evoked Ca^{2+} transients of abJGCs residing in the contralateral control and ipsilateral odor-deprived hemibulbs. (B-D) Box plots illustrating the fractions of odor responding cells per mouse (B) and (per cell) the amplitude (C) and AUC (D) of odor-evoked Ca^{2+} transients. n=13/4 and 29/4 cells/mice for contralateral control and ipsilateral odor-deprived hemibulbs. Data are shown as median ± IQR. **$P<0.01$.

Supplementary Figure S7. Immunohistochemical analyses confirming the retarded morphology of Kv1.2- and Kir2.1-expressing adult-born JGCs. (A) Sample images of abJGCs (20 dpi) belonging to control, Kv1.2 and Kir2.1 groups, reconstructed from *in vitro* images taken in 50 µm thick fixed slices,

immunofluorescently labeled against Twitch-2B. (B) Box plots showing (per cell) the TDBL of abJGCs. (C) Box plots showing (per cell) the number of dendritic branches of abJGCs. (B) and (C): n=26/5, 25/5, and 25/5 cells/mice for control, Kv1.2, and Kir2.1, respectively. (D) Sholl analysis illustrating the complexity of adult-born JGC's dendritic morphology in fixed brain slices at 20 dpi. n=25/5 cells/mice per group. (B) and (C): Data are shown as median ± IQR. (D) Data are shown as mean ± SEM. **P*<0.05, ***P*<0.01, ****P*<0.001, ns=not significant.

Supplementary Figure S8. Expression of Kir2.1 channel with a dominant-negative loss-of-function mutation (Kir2.1mut) did not impair the morphogenesis of adult-born JGCs. (A) Sample reconstructions of *in vivo* abJGCs (20 dpi) expressing Kir2.1mut. (B) Box plots showing (per cell) the TDBL of abJGCs imaged *in vivo* at 20 dpi in control, Kir2.1, and Kir2.1mut groups. (C) Box plots showing (per cell) the number of dendritic branches of abJGCs imaged *in vivo* at 20 dpi in control, Kir2.1, and Kir2.1mut groups. Control and Kir2.1 datasets are the same as in Figure 2. (D) Sholl analysis of Kir2.1- and Kir2.1mut-expressing abJGCs. (E) Sholl analysis of control and Kir2.1mut-expressing abJGCs. (D) and (E): n=33/7, 46/6, and 35/3 cells/mice for control, Kir2.1, and Kir2.1mut groups. (B) and (C): Data are shown as median ± IQR. (D) and (E): Data are shown as mean ± SEM. ****P*<0.001, ns=not significant. Note that Kir2.1mut was overexpressed using a retrovirus. The lack of functional difference between the retro- and lentivirus-infected neuronal progenitors is documented by the high similarity of their migration properties (compare Fig. 1 of the present study with Fig. 4 in ref. (2)).

Supplementary Figure S9. Neither Kv1.2 or Kir2.1 overexpression nor odor deprivation affected basal and maximum Twitch-2B ratios and AUCs of adult-

born JGCs. Box plots showing the median (per cell) basal and maximum Twitch-2B ratios and AUCs of spontaneous Ca^{2+} transients in abJGCs in control, Kv1.2, and Kir2.1 groups (A-C) as well as contralateral control and ipsilateral odor-deprived groups (D-F). n=60/8, 68/7, 66/5, 34/4, and 47/4 cells/mice for control, Kv1.2, Kir2.1, contralateral control, and ipsilateral odor-deprived groups, respectively. Data are shown as median ± IQR. ns=not significant.

Supplementary Figure S10. Relative expression levels of transcripts involved in pathways related to neuronal development, differentiation, and cell death. Bar graphs showing mean relative expression levels of mRNA encoding for the calcium/calmodulin-dependent protein kinase II gamma (*Camk2g*; A), doublecortin (*Dcx*; B), microtubule-associated protein tau (*Mapt*; C), neural cell adhesion molecule 1 (*Ncam1*; D), synaptophysin (*Syp*; E), BH3 interacting domain death agonist (*Bid*; F) and ribosomal protein L5 (*Rpl5*; G), as determined by qPCR analyses of FACS-sorted adult-born cells (see SI Materials and Methods). Data are shown as mean ± SEM. n = 3 biological replicates. $^{*}P < 0.05$, $^{**}P<0.01$, $^{***}P<0.001$, ns=not significant.

Supplementary Figure S11. Morphology and relative pCREB levels of adult-born granule cells from control, Kv1.2, and Kir2.1 groups. (A) Sample images of adult-born GCs (20 dpi) belonging to control, Kv1.2 and Kir2.1 groups, reconstructed from *in vitro* images taken in 50 µm thick fixed slices, immunofluorescently labeled against Twitch-2B. (B) Box plots showing (per cell) the TDBL (B) and the number of dendritic branches (C) of adult-born GCs. (D) Sholl analysis illustrating the complexity of adult-born GCs' dendritic morphology in fixed brain slices at 20 dpi. (E) Sample MIP images (15 µm depth) showing pCREB-, Twitch-2B- and NeuN-positive cells in the granule cell layer of the OB slices from control, Kv1.2 and Kir2.1 groups at 10 dpi. Twitch-2B labels

the adult-born GCs whereas NeuN labels the mature neurons. Arrowheads highlight the location of abJGCs. (F) Box plots illustrating the median (per mouse) relative pCREB levels of adult-born GCs in control, Kv1.2, and Kir2.1 groups at 10 dpi. n=119/5, 207/4, and 222/5 cells/mice for control, Kv1.2 and Kir2.1, respectively. (B-D): n=177/7, 149/5, and 133/6 cells/mice for control, Kv1.2 and Kir2.1 respectively. (B) (C) and (F): Data are shown as median ± IQR. (D): Data are shown as mean ± SEM. *$P<0.05$, ***$P<0.001$, ns=not significant.

Supplementary Figure S12. Relative expression levels of transcripts encoding for N-methyl-D-aspartate (NMDA) receptors and members of mitogen-activated protein kinase (MAPK) signaling cascade. Box plots showing mean values of z-scores for four isoforms of the NMDA receptor (A) and 18 members of the MAPK/ERK signaling cascade (B). All transcripts included in the box plots are listed in Table S1. n = 2 biological replicates (12 mice in total, 2 mice per replicate per group). Data are shown as median ± IQR. **$P<0.01$, ***$P<0.001$, ns=not significant.

Supplementary Table S1. Transcripts included in the box plots shown in Figures 7 and S11.

Figure	Genes
7B	*Camk2g, Camk2b, Camk2a, Calm3, Cacna1c, Cacna1d*
7C	*Vgefa, Nr4a1, Egr1, Negr1, Fra2 (from cFos)*
7D	*Camk2a, Camk2b, Cck, Dclk1, Dcx, Nr2f1, Fyn,Fzd3, Gja1,Mdk, Mapt, Ntrk2, Ptprz1, Reln, Olfm1, Kirrel3, Fbxo31, Gpm6a, Mrtfb, Adgrl3, Cdkl5, Flrt2*
7E	*Bdnf, Camk2b,Cdh2,Cntn1,Dcx,Nr2f1,Fyn,Grin1,Id4,Itsn1,Mdk, Meis1, Map1b, Map2, Mapt, Ncoa1, Nedd4, Nefl, Nf1, Ntrk2, Pcp4, Ptn, Ptprs, Ptprz1, Reln, Snap25, Snap91, Plk2, Sox9, Syt1, Dpysl3, Vim, Ywhag, Magi2, Olfm1, Rapgef4, Syne1, Fuom, Grip1, Plppr5, Fbxo31, Kank1, Baiap2, Eif4g1, Gpr37l1, Cpeb3, Adgrb3, Cdkl3, Arhgap44, Acsl6, Arhgap33, Amigo1, Ndrg4, Ntm, Fez1, Shank1, Nlgn3, Robo2, Nrcam, Il1rapl1, Cdkl5, Kalrn, Tnik, Acp4*
7F	*Camk2a, Camk2b, Dclk1, Dcx, Epha5, Fyn, Grin1, Nedd4, Ctnnd2, Ptn, Ptprz1, Reln, Syne1, Fbxo31, Baiap2, Adgrb3, Cdkl3, Arhgap44, Arhgap33, Shank1, Nlgn3, Il1rapl1, Cdkl5, Kalrn, Tnik*
7G	*Bcl10, Bcl7a, Bad, Casp8, Casp6, Casp1, Casp4, Casp12, Casp7, Casp2, Bcl2a1a, Bax, Bid*
7H	*Psmc5, Gapdh, Mkrn1, Tuba1b, Dars, Rpl27, Ldha, Rpl5, Actb*
S11A	*Nmda2b, Nmda2a, Nmda1, Nmda3a*
S11B	*Mapk1, Mapk2, Mapk3, Mapk6, Mapk7, Mapk8, Mapk9, Mapk10, Mapk14, Lamtor1, Lamtor2, Lamtor3, Lamtor4, Lamtor5, Mapkapk3, Mapkap1, Mapk1ip1, Jkamp*

Supplementary Table S2. Differential expression of factors interacting with CREB

Genes	Control vs Kv1.2		Control vs Kir2.1	
	logFC	*P* value	logFC	*P* value
Crtc2	4.59	0.1818	6.34	0.1002
Crtc3	0.21	0.9484	0.61	0.8533
Lmo4	-1.65	0.2554	-1.04	0.4539
Dream (Kcnip3)	-0.84	0.5959	-8.61	0.0046
Crest (Ss18l1)	-6.04	0.1098	0.56	0.8377
Smarca4	-1.55	0.5495	-0.91	0.7210
Hdac1	-1.84	0.3210	0.29	0.8663

Supplementary Table S3. Primers used for qPCR

Gene	Accession N°	Primers	Size (bp)
Gapdh	NM_001289726.1	F: TGCACCACCAACTGCTTAG<br>R: GGATGCAGGGATGATGTTC	177
Rpl5	NM_016980.2	F: TGGCAGACTACATGCGCTAC<br>R: ATACATCTCCTCCATCATGTCTG	109
Kcna2	NM_008417.5	F: TGGTACCCATCTGCAAGGGC<br>R: CCTTTGGAAGGAAGGAGGCAA	99
Kcnj2	NM_008425.4	F: GGCAAGCAGTGTCTTGGGAAT<br>R: AGAGAACTTGTCCTGTTGCTGG	138
Dcx	NM_001110222.1	F: GAGTGGGGCTTTCGAGTGAT<br>R: AAAGAAAGCCGTGTGCCTTG	78
Camk2g	NM_178597.5	F: AGTCAGATGGCGGTGTCAAG<br>R: GCTGGGCTTACGAGACTGTT	91
Mapt	NM_001038609.2	F: CGCCCCTAGTGGATGAGAGA<br>R: TCCTTCTGGGATCTCCGTGT	71
Ncam1	NM_001081445.1	F: CAAGTACAGAGCGCTCGCC<br>R: AGGGACTTGAGCATGACGTG	81
Syp	NM_009305.2	F: CTGCGTTAAAGGGGGCACTA<br>R: GGAAGTCCATCATTGGCCCT	175
Bid	NM_007544.4	F: GAGTGTGGCTCCGCAAACC<br>R: AGGGAATCACCACGCAGACG	78

mRNA sequence accession numbers were obtained from the Mouse Genome Database accessed at www.ncbi.nlm.nih.gov/genome.

Supplementary Table S4. Exact results of all statistical tests

Figure	Statistical test	*Post hoc* comparisons
1C	Kruskal-Wallis test: $P=2\times10^{-4}$	Dunn's multiple comparison test: control vs. Kv1.2: $P=0.04$, control vs. Kir2.1: $P=2\times10^{-4}$, Kv1.2 vs. Kir2.1: $P=0.22$.

1D	Kruskal-Wallis test: P=6×10^{-5}	Dunn's multiple comparison test: control vs. Kv1.2: P=2×10^{-4}, control vs. Kir2.1: P=1.6×10^{-3}, Kv1.2 vs. Kir2.1: P=0.36.
1E	Kruskal-Wallis test: P=2×10^{-4}	Dunn's multiple comparison test: control vs. Kv1.2: P=3×10^{-4}, control vs. Kir2.1: P=6.3×10^{-3}, Kv1.2 vs. Kir2.1: P=0.67.
1F	Kruskal-Wallis test: P=3×10^{-4}	Dunn's multiple comparison test: control vs. Kv1.2: P=8.9×10^{-3}, control vs. Kir2.1: P=3×10^{-3}, Kv1.2 vs. Kir2.1: P=0.55.
1G	One-way ANOVA: $F_{2,19}$=7.982, P=3×10^{-3}	Tukey's multiple comparison test: control vs. Kv1.2: P=0.01, control vs. Kir2.1: P=6.6×10^{-3}, Kv1.2 vs. Kir2.1: P=0.59.
1I, M	Paired *t* test, P>0.05 for all comparisons	
1J-L	Two-sided unpaired *t* test, P>0.05 for all comparisons.	For the 4 paired olfactory bulbs: Paired *t* test, P>0.05 for all comparisons
2B	Kruskal-Wallis test: P=2×10^{-9}	Dunn's multiple comparison test: control vs. Kv1.2: P=3×10^{-9}; control vs. Kir2.1: P=8×10^{-6}; Kv1.2 vs. Kir2.1: P=0.21
2C	Kruskal-Wallis test: P=5×10^{-10}	Dunn's multiple comparison test: control vs. Kv1.2: P=2×10^{-9}; control vs. Kir2.1: P=5×10^{-7}; Kv1.2 vs. Kir2.1: P=0.58
2D	Generalized linear mixed model fit by maximum likelihood (Laplace Approximation)	control vs. Kv1.2: P<0.001, control vs. Kir2.1: P<0.001, Kv1.2 vs. Kir2.1: P<0.001
2F, G	Paired *t* test, P>0.05 for both comparisons	
2H	Generalized linear mixed model fit by maximum likelihood (Laplace Approximation)	P=0.98
3B	One-way ANOVA: $F_{2,15}$=6.747, P=8.1×10^{-3}	Tukey's multiple comparison test: control vs. Kv1.2: P=0.02; control vs. Kir2.1: P=9.6×10^{-3}; Kv1.2 vs. Kir2.1: P=0.69.
3D	One-way ANOVA: $F_{2,11}$=13.84, P=1×10^{-3}	Tukey's multiple comparison test: control vs. Kv1.2: P=0.81; control vs. Kir2.1: P=1.2×10^{-3}; Kv1.2 vs. Kir2.1: P=3.3×10^{-3}.
4A	One-way ANOVA: $F_{2,16}$=7.061, P=0.0063	Tukey's multiple comparison test: control vs. Kv1.2: P=0.54; control vs. Kir2.1: P=5.9×10^{-3}; Kv1.2 vs. Kir2.1: P=0.04.

4C	Kruskal-Wallis test: *P*=2×10^{-6}	Dunn's multiple comparison test: control vs. Kv1.2: *P*=4×10^{-5}; control vs. Kir2.1: *P*=6×10^{-4}; Kv1.2 vs. Kir2.1: *P*>0.99.
4D	Kruskal-Wallis test: *P*=7×10^{-9}	Dunn's multiple comparison test: control vs. Kv1.2: *P*=3×10^{-6}; control vs. Kir2.1: *P*=4×10^{-6}; Kv1.2 vs. Kir2.1: *P*=0.39.
5B	Chi-square test, *P*=3×10^{-6}	
5C	One-way ANOVA: $F_{2,15}$=5.468, *P*=0.016	Tukey's multiple comparison test: control vs. Kv1.2: *P*=0.03; control vs. Kir2.1: *P*=6.2×10^{-3}; Kv1.2 vs. Kir2.1: *P*=0.57.
5E	Chi-square test, *P*=0.28	
5F	Paired *t* test, *P*=0.79	
6B	One-way ANOVA: $F_{2,14}$=45.73, *P*=7×10^{-7}	Tukey's multiple comparison test: control vs. Kv1.2: *P*=2×10^{-6}; control vs. Kir2.1: *P*=4×10^{-6}; Kv1.2 vs. Kir2.1: *P*=0.99.
6C	Paired *t*-test, *P*=0.57	
7A	One-way ANOVA: $F_{2,10}$=14.12, *P*=2.4×10^{-3}	Tukey's multiple comparison test: control vs. Kv1.2: *P*=2.9×10^{-3}; control vs. Kir2.1: *P*=2.2×10^{-3}; Kv1.2 vs. Kir2.1: *P*=0.99.
7B	One-way ANOVA: $F_{2,14}$=18.02, *P*=1.3×10^{-4}	Tukey's multiple comparison test: control vs. Kv1.2: *P*=4.3×10^{-3}; control vs. Kir2.1: *P*=1.1×10^{-4}; Kv1.2 vs. Kir2.1: *P*=0.15.
7C	One-way ANOVA: $F_{2,11}$=9.768, *P*=3.6×10^{-3}	Tukey's multiple comparison test: control vs. Kv1.2: *P*=1.5×10^{-2}; control vs. Kir2.1: *P*=4.6×10^{-3}; Kv1.2 vs. Kir2.1: *P*=0.89.
7D	Friedman test, *P*=1×10^{-15}	Dunn's multiple comparison test: control vs. Kv1.2: *P*=1×10^{-15}; control vs. Kir2.1: *P*=1×10^{-15}; Kv1.2 vs. Kir2.1: *P*=0.75.
7E	One-way ANOVA: $F_{2,48}$=99.8, *P*=1×10^{-15}	Tukey's multiple comparison test: control vs. Kv1.2: *P*=1×10^{-15}; control vs. Kir2.1: *P*=2×10^{-15}; Kv1.2 vs. Kir2.1: *P*=0.84.
7F	One-way ANOVA: $F_{2,26}$=22.02, *P*=2.5×10^{-6}	Tukey's multiple comparison test: control vs. Kv1.2: *P*=1.3×10^{-5}; control vs. Kir2.1: *P*=1.4×10^{-5}; Kv1.2 vs. Kir2.1: *P*=0.99.
7G	One-way ANOVA: $F_{2,16}$=1.27, *P*=0.31	
S1C	Kolmogorov-Smirnov test, *P*=1×10^{-8}, *D*=0.399.	
S1D	Unpaired *t* test with Welch's correction, t_5 =17.13, *P*=1×10^{-5}.	

S1E	One-way ANOVA: $F_{2,6}$=696.8, *P*=7.9×10^{-8}	Tukey's multiple comparison test: control vs. Kv1.2: *P*=2.4×10^{-7}; control vs. Kir2.1: *P*=0.98; Kv1.2 vs. Kir2.1: *P*=2.5×10^{-7}.
S1F	One-way ANOVA: $F_{2,6}$=8.16, *P*=0.02	Tukey's multiple comparison test: control vs. Kv1.2: *P*=0.97; control vs. Kir2.1: *P*=0.03; Kv1.2 vs. Kir2.1: *P*=0.03.
S2B	Two-sided unpaired *t* test, *P*=0.26	
S2C	Two-sided unpaired *t* test, *P*=0.26	
S2D	Two-sided unpaired *t* test, *P*=0.77	
S2E	Two-sided unpaired *t* test, *P*=0.78	
S2F	Two-sided paired *t* test, *P*<0.001	
S2G	Two-sided unpaired *t* test, *P*=0.04	
S3C	One-way ANOVA: $F_{2,12}$=0.943, *P*=0.416	
S3D	One-way ANOVA: $F_{2,12}$=0.95, *P*=0.415	
S4A	One-way ANOVA: $F_{2,17}$=0.05, *P*=0.95	
S4B	One-way ANOVA: $F_{2,13}$=2.18, *P*=0.15	
S5A	One-way ANOVA: $F_{2,18}$=4.01, *P*=0.04	unpaired t test with Welch's correction for multiple comparisons: control vs. Kv1.2: *P*=0.17; control vs. Kir2.1: *P*=1.9×10^{-3}; Kv1.2 vs. Kir2.1: *P*=0.04.
S5B	Two-sided Mann-Whitney test, *P*=0.68	
S5C	Two-sided Mann-Whitney test, *P*=0.82	
S5D	Two-sided Mann-Whitney test, *P*=0.02	
S6B	Unpaired *t* test with Welch's correction, t_3=6.31, *P*=8×10^{-3}	
S7B	Kruskal-Wallis test: *P*=5×10^{-9}	Dunn's multiple comparison test: control vs. Kv1.2: *P*=5×10^{-9}; control vs. Kir2.1: *P*=4×10^{-6}; Kv1.2 vs. Kir2.1: *P*=0.72

S7C	Kruskal-Wallis test: P=6×10^{-10}	Dunn's multiple comparison test: control vs. Kv1.2: P=1×10^{-9}; control vs. Kir2.1: P=2×10^{-5}; Kv1.2 vs. Kir2.1: P=0.27
S7D	Generalized linear mixed model fit by maximum likelihood (Laplace Approximation)	control vs. Kv1.2: P=0.004, control vs. Kir2.1: P=0.011, Kv1.2 vs. Kir2.1: P=0.016
S8B	Kruskal-Wallis test: P=1×10^{-9}	Dunn's multiple comparison test: control vs. Kir2.1: P=1×10^{-5}; control vs. Kir2.1mut: P=0.7; Kir2.1 vs. Kir2.1mut: P=6×10^{-9}
S8C	Kruskal-Wallis test: P=7×10^{-11}	Dunn's multiple comparison test: control vs. Kir2.1: P=3×10^{-7}; control vs. Kir2.1mut: P=0.99; Kir2.1 vs. Kir2.1mut: P=3×10^{-9}
S8D	Generalized linear mixed model fit by maximum likelihood (Laplace Approximation)	P<0.001
S8E	Generalized linear mixed model fit by maximum likelihood (Laplace Approximation)	P=0.28
S9A-C	Kruskal-Wallis test, P>0.05 for all tests	
S9D-F	Mann-Whitney test, P>0.05 for all tests	
S10A	One-way ANOVA: $F_{2,6}$=25.21, P=0.0012	Fisher's multiple comparison test: control vs. Kv1.2: P=8.2×10^{-4}; control vs. Kir2.1: P=8.8×10^{-4}; Kv1.2 vs. Kir2.1: P=0.94.
S10B	One-way ANOVA: $F_{2,6}$=10.77, P=0.01	Fisher's multiple comparison test: control vs. Kv1.2: P=8.3×10^{-3}; control vs. Kir2.1: P=6×10^{-3}; Kv1.2 vs. Kir2.1: P=0.78.
S10C	One-way ANOVA: $F_{2,6}$=27.17, P=9.8×10^{-4}	Fisher's multiple comparison test: control vs. Kv1.2: P=6.5×10^{-4}; control vs. Kir2.1: P=7.4×10^{-4}; Kv1.2 vs. Kir2.1: P=0.88.
S10D	One-way ANOVA: $F_{2,6}$=43.04, P=2.7×10^{-4}	Fisher's multiple comparison test: control vs. Kv1.2: P=1.9×10^{-4}; control vs. Kir2.1: P=2.1×10^{-4}; Kv1.2 vs. Kir2.1: P=0.91.
S10E	One-way ANOVA: $F_{2,6}$=46.98, P=2.2×10^{-4}	Fisher's multiple comparison test: control vs. Kv1.2: P=1.6×10^{-4}; control vs. Kir2.1: P=1.5×10^{-4}; Kv1.2 vs. Kir2.1: P=0.89.

S10F	One-way ANOVA: $F_{2,6}$=9.19, *P*=0.015	Fisher's multiple comparison test: control vs. Kv1.2: *P*=6×10^{-3}; control vs. Kir2.1: *P*=0.03; Kv1.2 vs. Kir2.1: *P*=0.24.
S10G	One-way ANOVA: $F_{2,6}$=0.15, *P*=0.86	
S11B	Kruskal-Wallis test: *P*=1×10^{-14}	Dunn's multiple comparison test: control vs. Kv1.2: *P*=1×10^{-14}; control vs. Kir2.1: *P*=1×10^{-14}; Kv1.2 vs. Kir2.1: *P*=0.99
S11C	Kruskal-Wallis test: *P*=1×10^{-14}	Dunn's multiple comparison test: control vs. Kv1.2: *P*=1×10^{-14}; control vs. Kir2.1: *P*=1×10^{-14}; Kv1.2 vs. Kir2.1: *P*=0.99
S11D	Generalized linear mixed model fit by maximum likelihood (Laplace Approximation)	control vs. Kv1.2: *P*<0.001, control vs. Kir2.1: *P*<0.001, Kv1.2 vs. Kir2.1: *P*<0.001
S11F	One-way ANOVA: $F_{2,11}$=6.524, *P*=0.013	Tukey's multiple comparison test: control vs. Kv1.2: *P*=0.02; control vs. Kir2.1: *P*=0.049; Kv1.2 vs. Kir2.1: *P*=0.69.
S12A	One-way ANOVA: $F_{2,6}$=31.74, *P*=6×10^{-4}	Tukey's multiple comparison test: control vs. Kv1.2: *P*=8×10^{-4}; control vs. Kir2.1: *P*=1.6×10^{-3}; Kv1.2 vs. Kir2.1: *P*=0.67.
S12B	One-way ANOVA: $F_{2,34}$=0.18, *P*=0.83	

Fig. S1

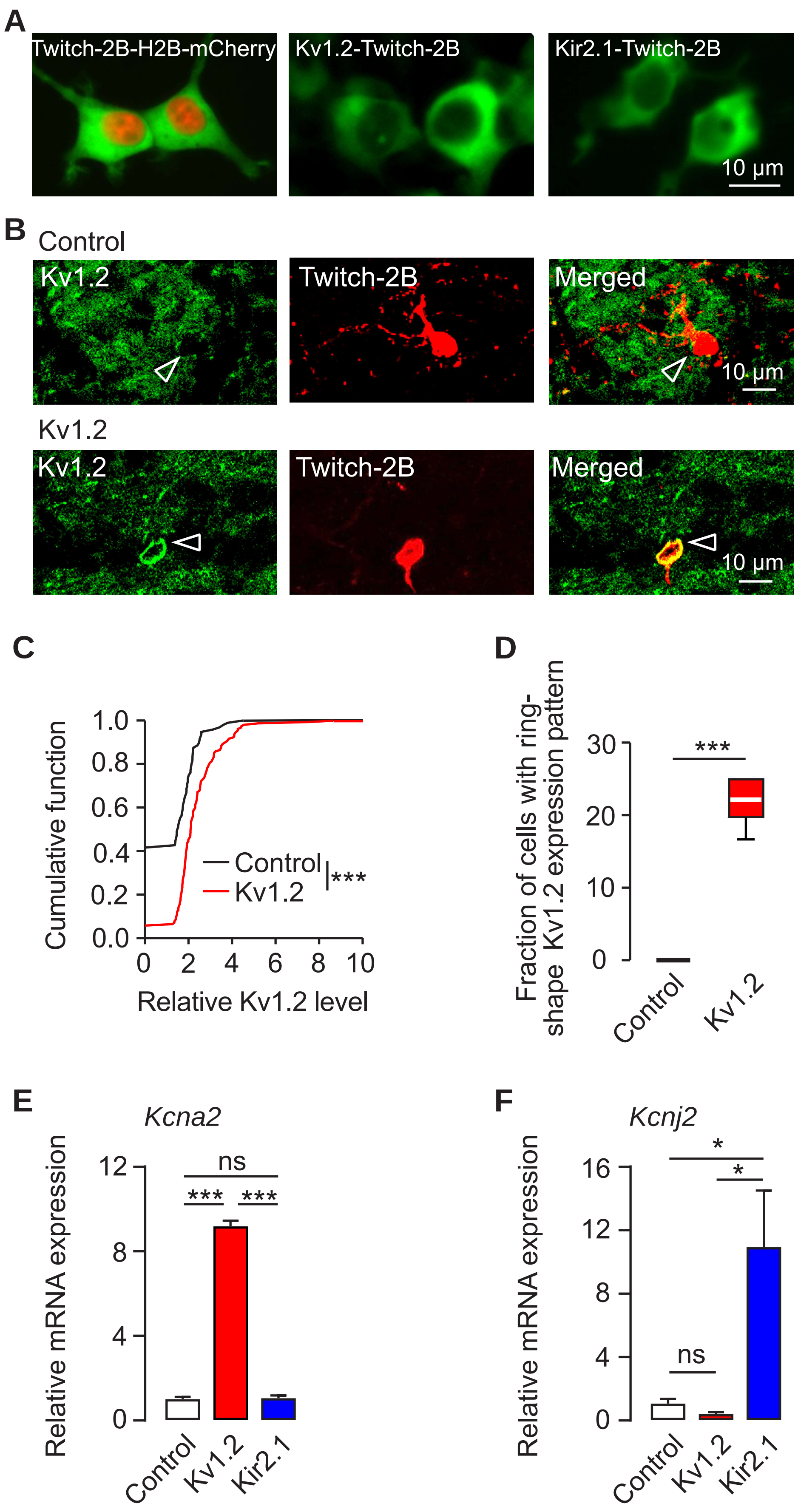

A
Twitch-2B-H2B-mCherry
Kv1.2-Twitch-2B
Kir2.1-Twitch-2B
10 µm
B
Control
Kv1.2
Twitch-2B
Merged
10 µm
Kv1.2
Kv1.2
Twitch-2B
Merged
10 µm
C
Cumulative function
1.0
0.8
0.6
0.4
0.2
0.0
0
2
4
6
8
10
Relative Kv1.2 level
Control
Kv1.2
***
D
Fraction of cells with ring-shape Kv1.2 expression pattern
30
20
10
0
***
Control
Kv1.2
E
Kcna2
Relative mRNA expression
12
8
4
0
ns
***
***
Control
Kv1.2
Kir2.1
F
Kcnj2
Relative mRNA expression
16
12
8
4
0
*
*
ns
Control
Kv1.2
Kir2.1

Fig. S2

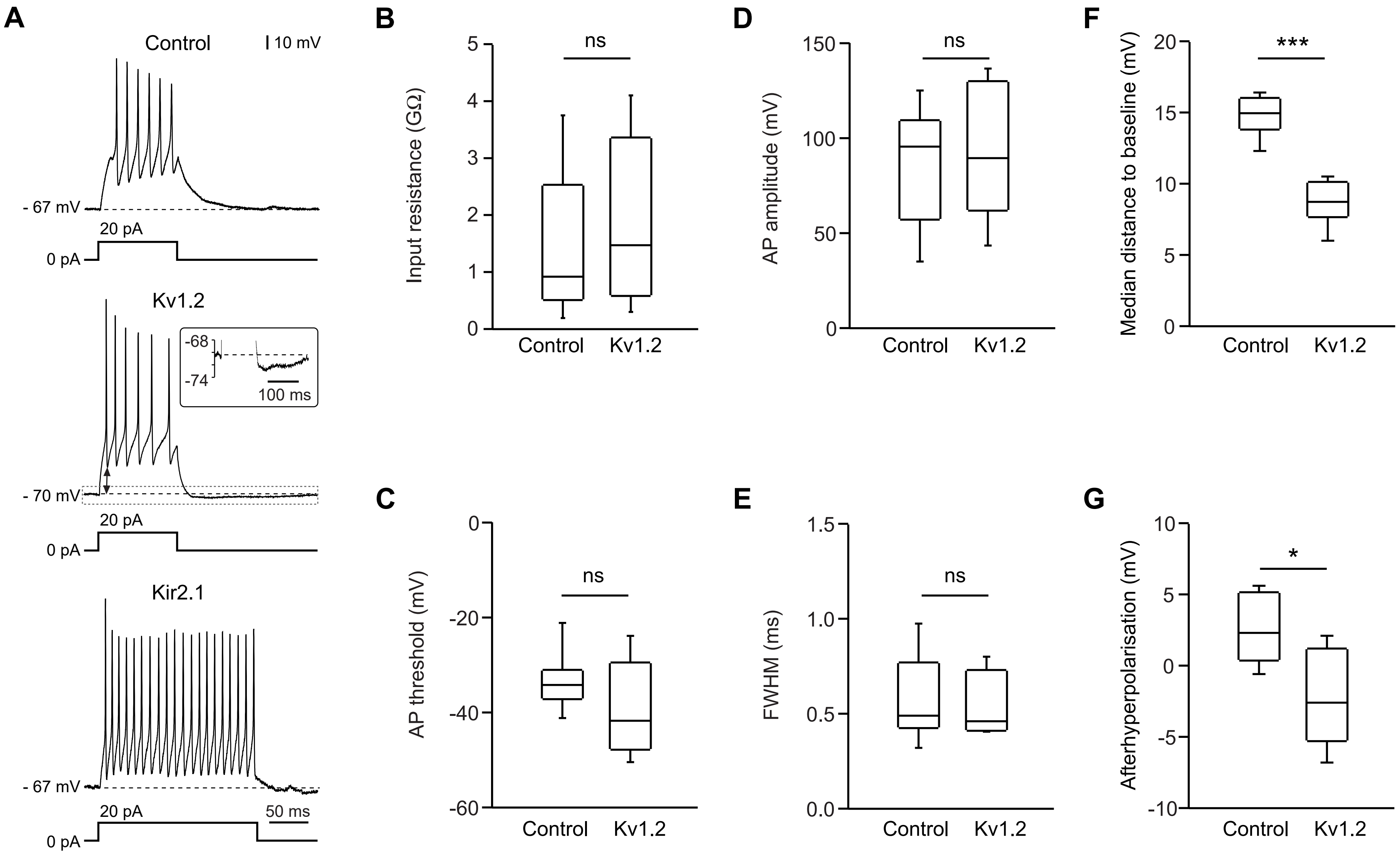
A
Control
10 mV
- 67 mV
20 pA
0 pA
Kv1.2
-68
-74
100 ms
- 70 mV
20 pA
0 pA
Kir2.1
- 67 mV
20 pA
50 ms
0 pA
B
Input resistance (GΩ)
ns
Control
Kv1.2
C
AP threshold (mV)
ns
Control
Kv1.2
D
AP amplitude (mV)
ns
Control
Kv1.2
E
FWHM (ms)
ns
Control
Kv1.2
F
Median distance to baseline (mV)
***
Control
Kv1.2
G
Afterhyperpolarisation (mV)
*
Control
Kv1.2

Fig. S3

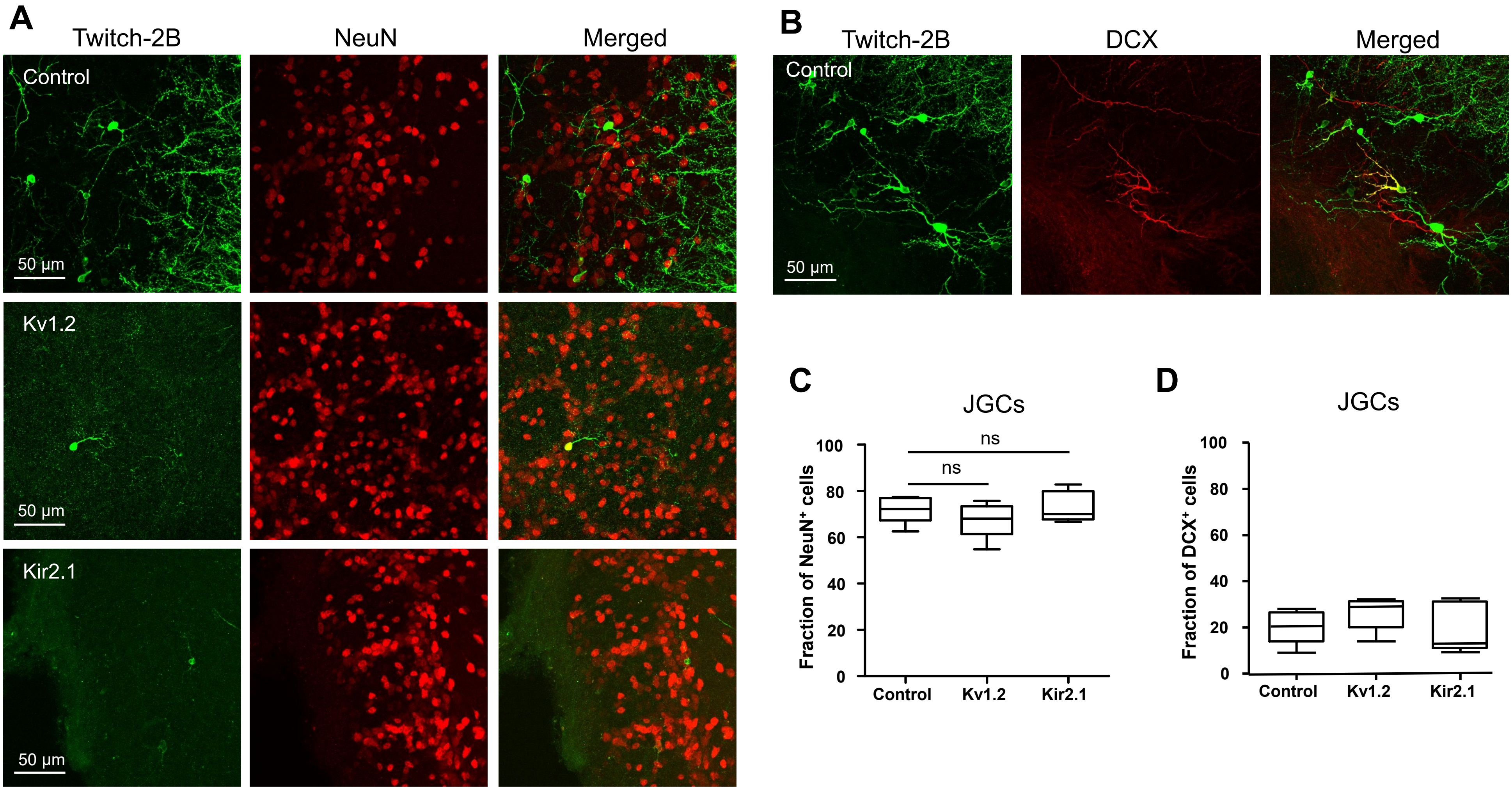
A
Twitch-2B
NeuN
Merged
Control
Kv1.2
Kir2.1
50 μm
B
Twitch-2B
DCX
Merged
Control
50 μm
C
JGCs
Fraction of NeuN+ cells
100
80
60
40
20
0
ns
ns
Control
Kv1.2
Kir2.1
D
JGCs
Fraction of DCX+ cells
100
80
60
40
20
0
Control
Kv1.2
Kir2.1

Fig. S4

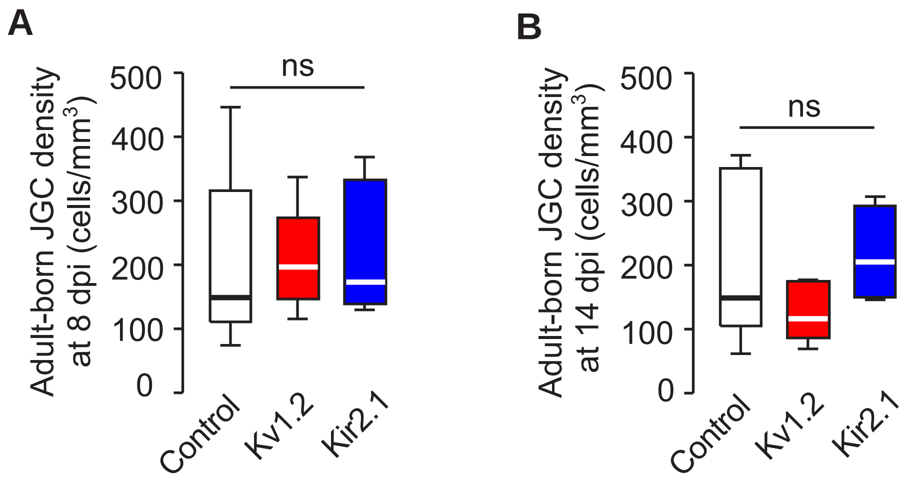
A
Adult-born JGC density at 8 dpi (cells/mm³)
500
400
300
200
100
0
ns
Control
Kv1.2
Kir2.1
B
Adult-born JGC density at 14 dpi (cells/mm³)
500
400
300
200
100
0
ns
Control
Kv1.2
Kir2.1

Fig. S5

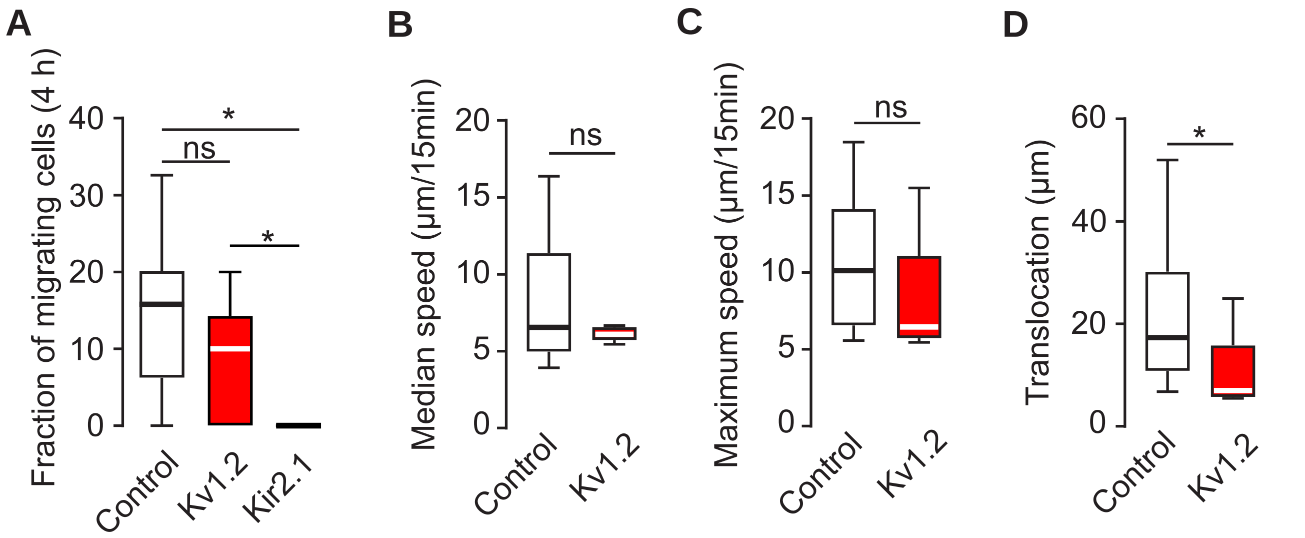
A
B
C
D
Fraction of migrating cells (4 h)
40
30
20
10
0
*
ns
*
Control
Kv1.2
Kir2.1
Median speed (µm/15min)
20
15
10
5
0
ns
Control
Kv1.2
Maximum speed (µm/15min)
20
15
10
5
0
ns
Control
Kv1.2
Translocation (µm)
60
40
20
0
*
Control
Kv1.2

Fig. S6

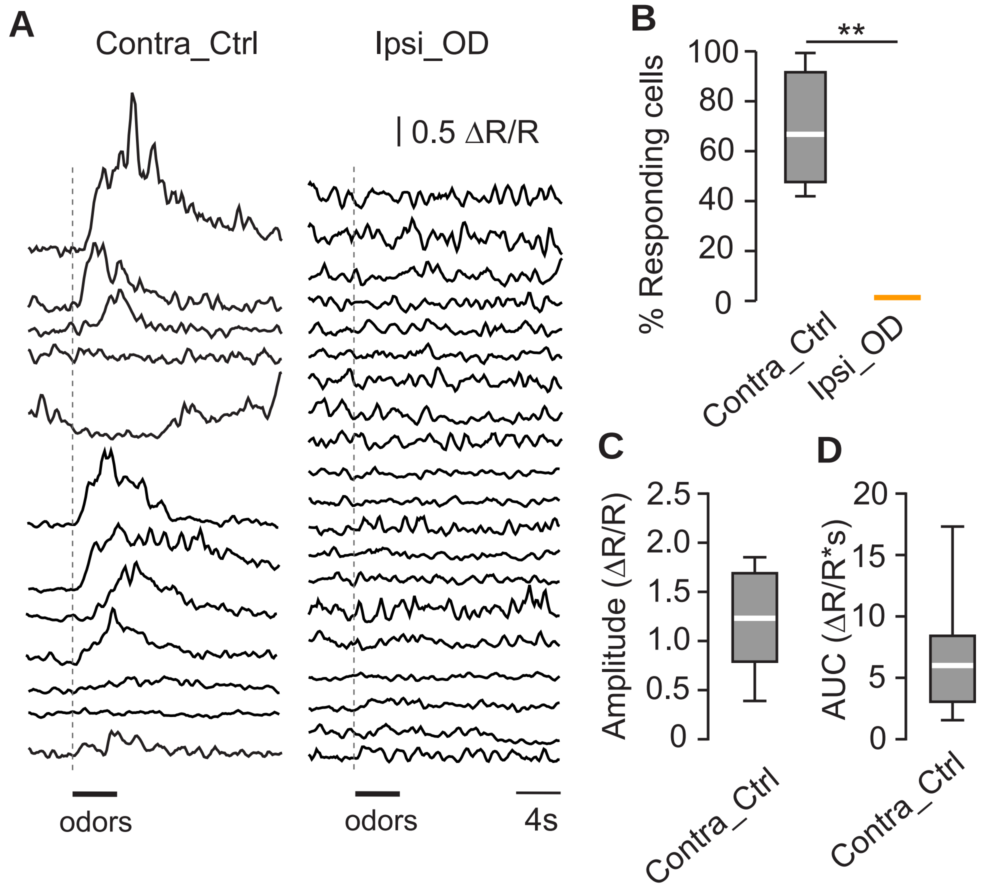
A
Contra_Ctrl
Ipsi_OD
| 0.5 ΔR/R
odors
odors
4s
B
**
% Responding cells
100
80
60
40
20
0
Contra_Ctrl
Ipsi_OD
C
Amplitude (ΔR/R)
2.5
2.0
1.5
1.0
0.5
0
Contra_Ctrl
D
AUC (ΔR/R*s)
20
15
10
5
0
Contra_Ctrl

Fig. S7

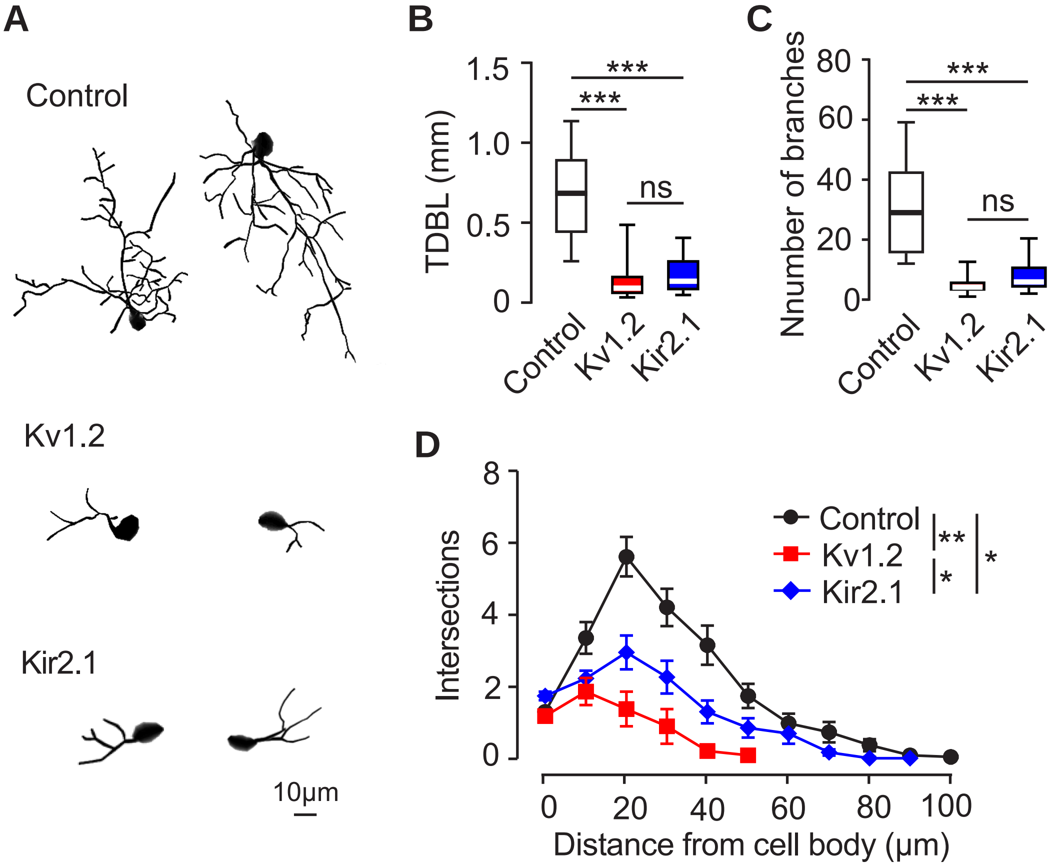
A
Control
Kv1.2
Kir2.1
10μm
B
TDBL (mm)
1.5
1.0
0.5
0
***
***
ns
Control
Kv1.2
Kir2.1
C
Nnumber of branches
80
60
40
20
0
***
***
ns
Control
Kv1.2
Kir2.1
D
Intersections
8
6
4
2
0
Control
Kv1.2
Kir2.1
**
*
*
0
20
40
60
80
100
Distance from cell body (μm)

Fig. S8

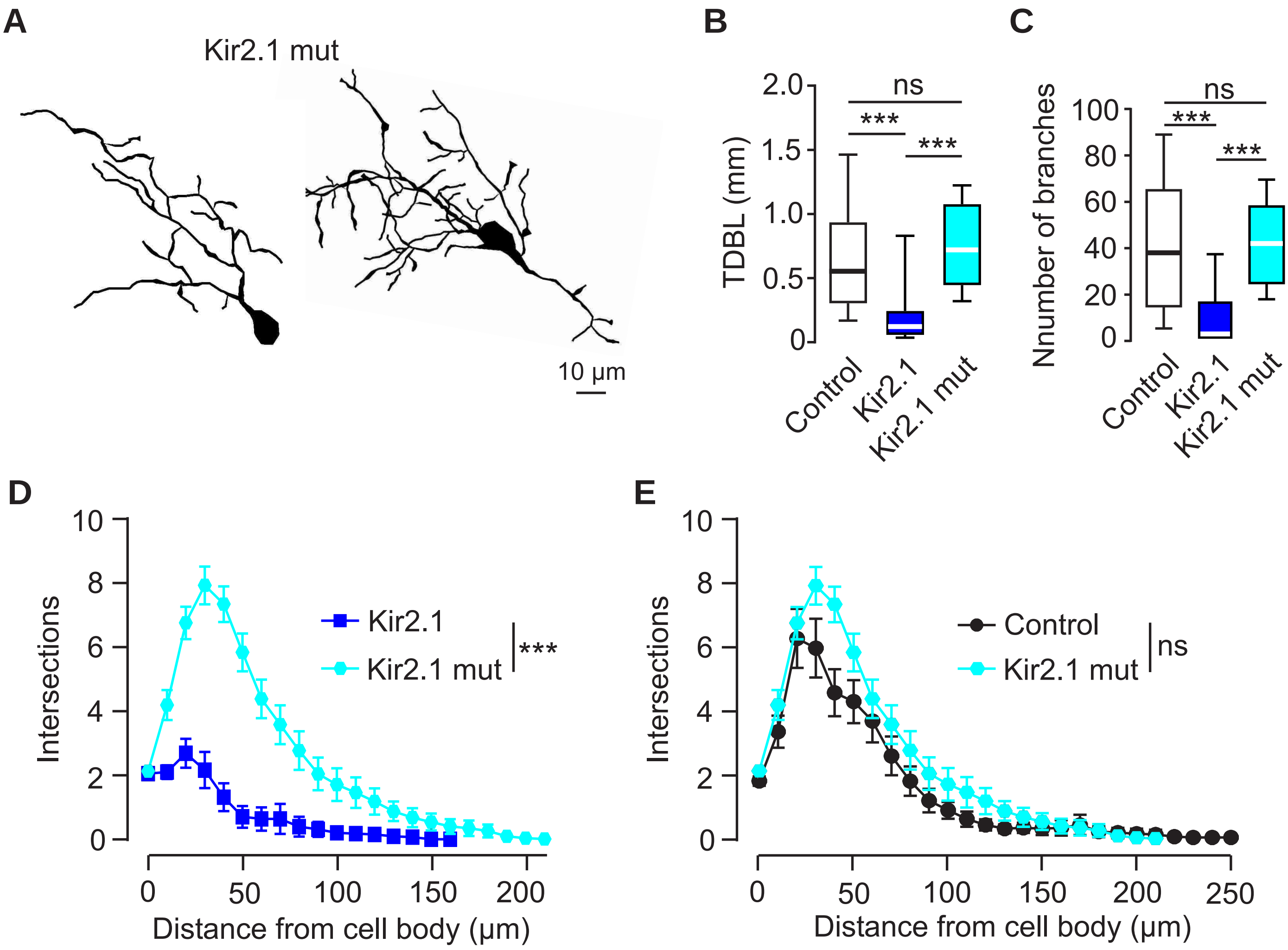
A
Kir2.1 mut
10 µm
B
TDBL (mm)
2.0
1.5
1.0
0.5
0
ns
***
***
Control
Kir2.1
Kir2.1 mut
C
Nnumber of branches
100
80
60
40
20
0
ns
***
***
Control
Kir2.1
Kir2.1 mut
D
Intersections
10
8
6
4
2
0
Kir2.1
Kir2.1 mut
***
0
50
100
150
200
Distance from cell body (µm)
E
Intersections
10
8
6
4
2
0
Control
Kir2.1 mut
ns
0
50
100
150
200
250
Distance from cell body (µm)

Fig. S9

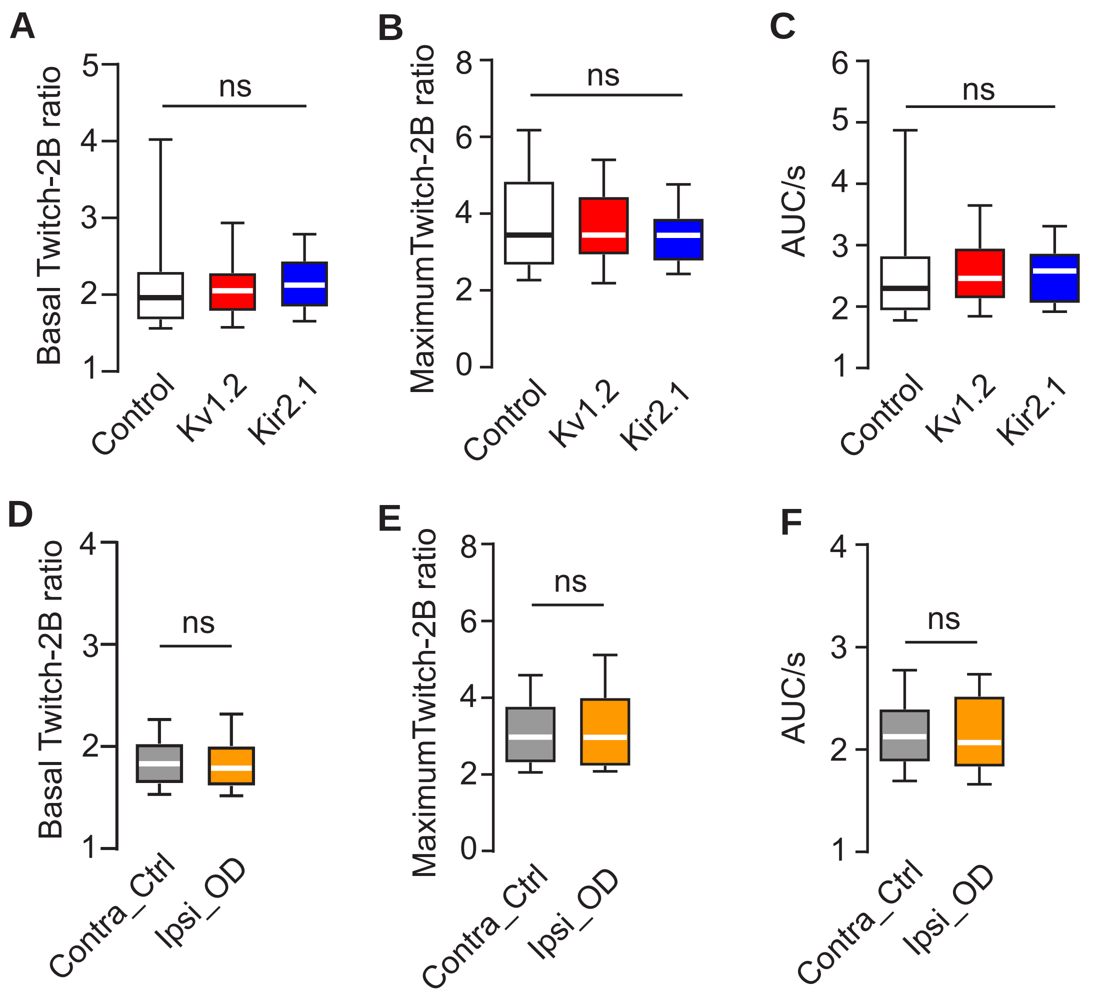
A
Basal Twitch-2B ratio
5
4
3
2
1
ns
Control
Kv1.2
Kir2.1
B
MaximumTwitch-2B ratio
8
6
4
2
0
ns
Control
Kv1.2
Kir2.1
C
AUC/s
6
5
4
3
2
1
ns
Control
Kv1.2
Kir2.1
D
Basal Twitch-2B ratio
4
3
2
1
ns
Contra_Ctrl
Ipsi_OD
E
MaximumTwitch-2B ratio
8
6
4
2
0
ns
Contra_Ctrl
Ipsi_OD
F
AUC/s
4
3
2
1
ns
Contra_Ctrl
Ipsi_OD

Fig. S10

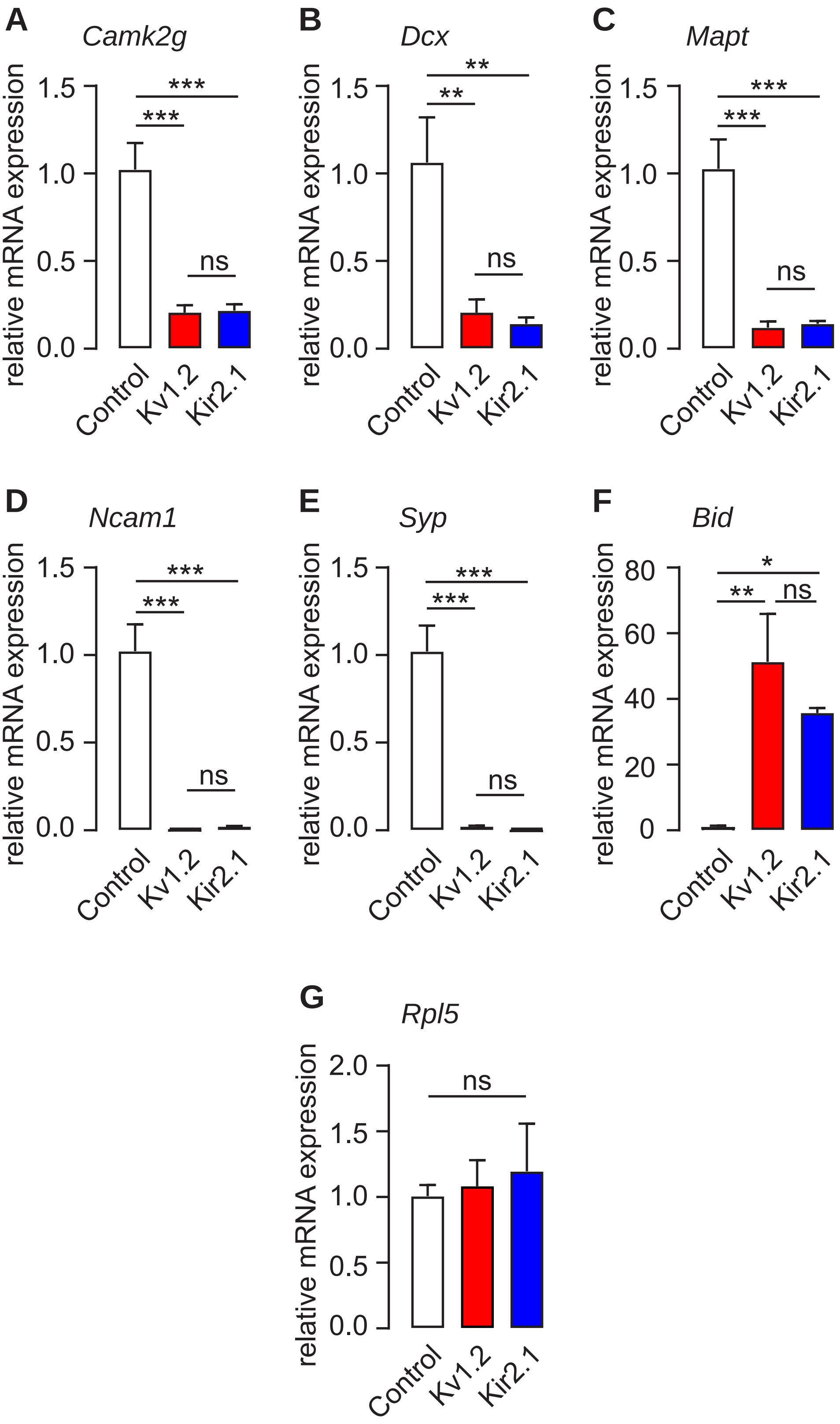

A
Camk2g
B
Dcx
C
Mapt
D
Ncam1
E
Syp
F
Bid
G
Rpl5
relative mRNA expression
Control
Kv1.2
Kir2.1
***
**
*
ns
0.0
0.5
1.0
1.5
2.0
0
20
40
60
80

Fig. S11

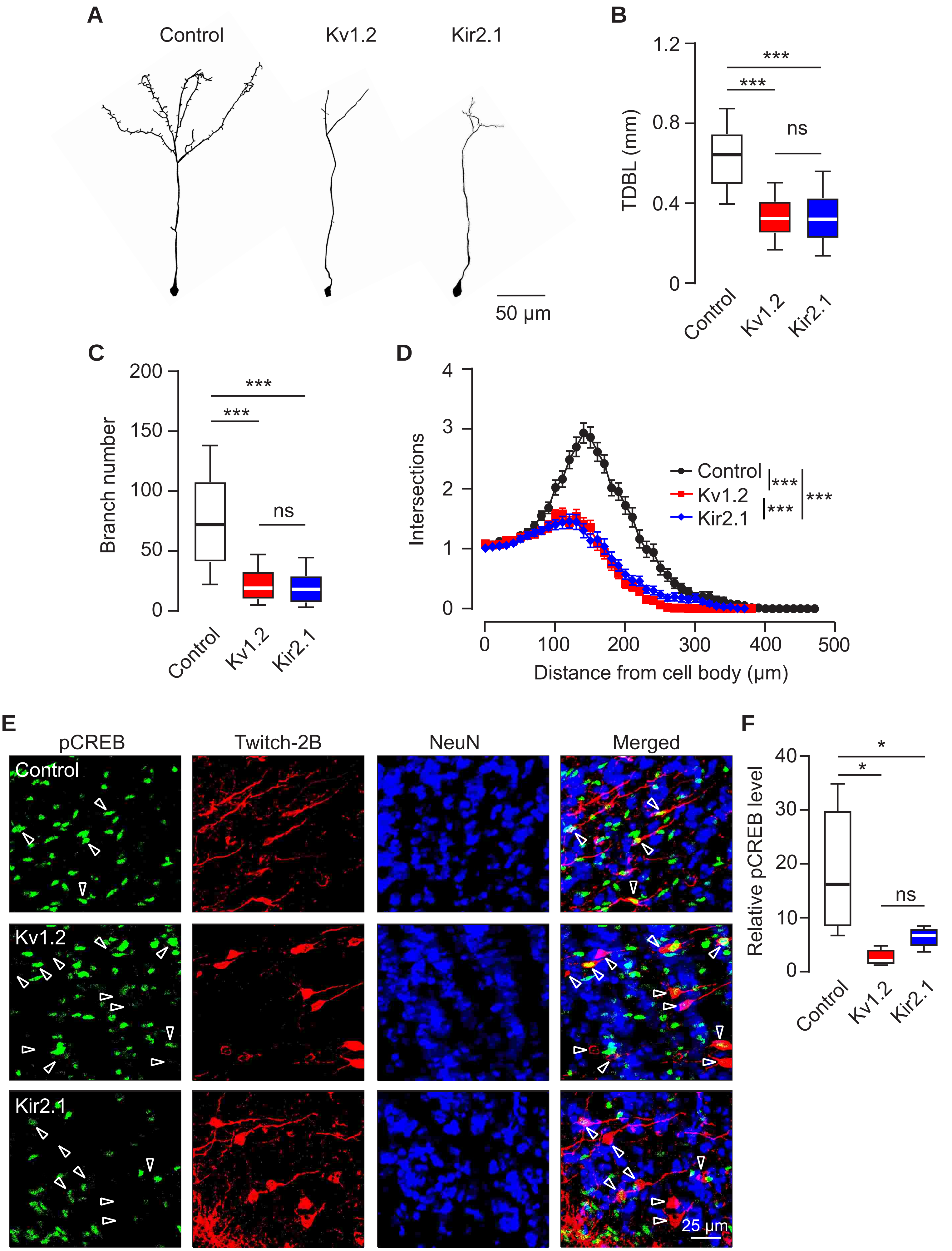
A
Control
Kv1.2
Kir2.1
50 μm
B
TDBL (mm)
1.2
0.8
0.4
0
***
***
ns
Control
Kv1.2
Kir2.1
C
Branch number
200
150
100
50
0
***
***
ns
Control
Kv1.2
Kir2.1
D
Intersections
4
3
2
1
0
Control
Kv1.2
Kir2.1
***
***
***
0
100
200
300
400
500
Distance from cell body (μm)
E
pCREB
Twitch-2B
NeuN
Merged
Control
Kv1.2
Kir2.1
25 μm
F
Relative pCREB level
40
30
20
10
0
*
*
ns
Control
Kv1.2
Kir2.1

Fig. S12

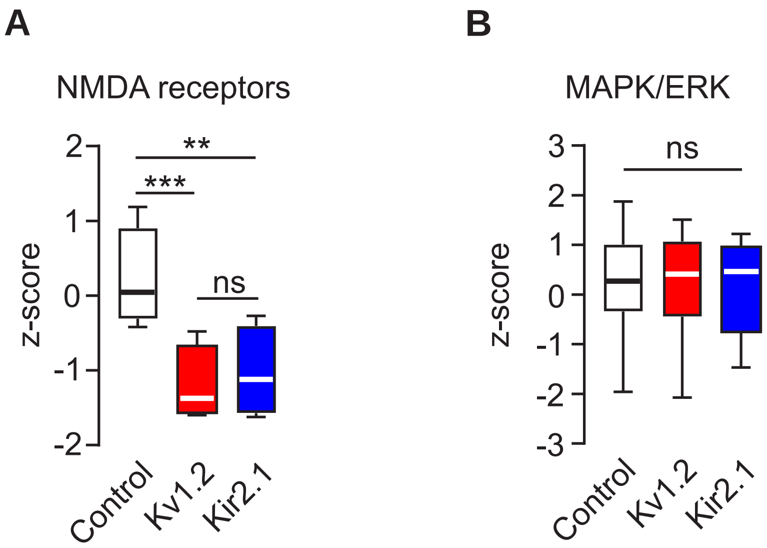
A
NMDA receptors
2
1
0
-1
-2
z-score
**
***
ns
Control
Kv1.2
Kir2.1
B
MAPK/ERK
3
2
1
0
-1
-2
-3
z-score
ns
Control
Kv1.2
Kir2.1
